# DNA methyltransferase inhibition is a therapeutic vulnerability in VHL-deficient renal cell carcinoma cells

**DOI:** 10.1038/s12276-026-01663-w

**Published:** 2026-03-06

**Authors:** Yue Pu, Ziruoyu Wang, Shishi Tao, Eun Ju Yang, Jin Zhang, Yu Han, Songlin Wu, Guowen Ren, Li-Jie Chen, Xiumei Zhang, Kaeling Tan, Gang Li, Kai Miao, Jianfeng Wang, Yongjun Dang, Joong Sup Shim

**Affiliations:** 1 Cancer Centre, Faculty of Health Sciences, University of Macau, Taipa, China; 2 Basic Medicine Research and Innovation Center for Novel Target and Therapeutic Intervention, Ministry of Education, College of Pharmacy, The Second Affiliated Hospital of Chongqing Medical University, Chongqing Medical University, Chongqing, China; 3 Institute of Cancer Research, Shenzhen Bay Laboratory, Shenzhen, China; 4 Department of Urology, Renji Hospital, Shanghai Jiaotong University, Shanghai, China; 5 Ministry of Education Frontiers Science Centre for Precision Oncology, University of Macau, Taipa, China

**Keywords:** Renal cell carcinoma, Targeted therapies

## Abstract

von Hippel–Lindau (VHL) is a tumor suppressor frequently inactivated in renal cell carcinoma (RCC), and its loss is associated with aberrant DNA methylation. Here we demonstrate that VHL-deficient RCC cells are highly vulnerable to DNA methyltransferase (DNMT) inhibitors. US Food and Drug Administration-approved DNMT inhibitors, such as decitabine and azacitidine, and investigational agents including RX-3117 and SGI-1027 selectively suppressed the growth of VHL-deficient RCC cells. Mechanistically, VHL loss leads to HIF-2α-dependent transcriptional upregulation of DNMT1, resulting in widespread CpG hypermethylation. Transcriptomic profiling and an RNA interference-based rescue screen identified KCNK3, a putative tumor suppressor, as a key mediator of DNMT inhibitor-induced synthetic lethality in VHL-deficient RCC. The KCNK3 promoter is hypermethylated and transcriptionally repressed in VHL-deficient RCC, where treatment with DNMT inhibitors reverses this methylation, restoring KCNK3 expression and resulting in cell growth inhibition. Silencing KCNK3 significantly attenuated the antitumor effects of DNMT inhibitors both in vitro and in vivo. Further mechanistic analysis showed that KCNK3 reactivation triggers TNF-α, MAPK and apoptotic signaling pathways, contributing to the observed synthetic lethality. Collectively, these findings establish DNMT inhibition as a synthetic lethal strategy in VHL-deficient RCC and highlight a potential therapeutic vulnerability for personalized treatment approaches.

## VHL loss creates sensitivity to DNMT inhibitors

This study explores how a specific gene, von Hippel–Lindau (VHL), affects DNA methylation in kidney cancer. Researchers found that when VHL is missing, certain cancer cells become more sensitive to drugs that inhibit DNA methylation. The study used various laboratory techniques to test this idea. They used cell lines and treated them with drugs such as decitabine and azacitidine, which are known to inhibit DNA methylation. These drugs were particularly effective in killing cancer cells lacking VHL. The researchers also tested these findings in mice and confirmed that the drugs slowed tumor growth without causing significant side effects. The study identified a specific gene, KCNK3, which is usually silenced in VHL-deficient cells but can be reactivated by these drugs. Researchers conclude that targeting DNA methylation could be a promising strategy for treating kidney cancer with VHL loss.

This summary was initially drafted using artificial intelligence, then revised and fact-checked by the author.

## Introduction

Renal cell carcinoma (RCC), the most common form of kidney cancer, originates from the epithelial cells of the renal tubules. It comprises several histological subtypes, including clear cell RCC (~70–80%), papillary RCC (~10–20%) and chromophobe RCC (<5%)^1,2^. Although early-stage RCC is associated with a high survival rate, it is often difficult to detect due to the absence of specific symptoms. By contrast, treatment of advanced or metastatic RCC remains challenging, as the disease tends to be resistant to conventional radiotherapy and chemotherapy^3,4^. Classical immunotherapeutic agents, such as interferon alpha (IFN-α) and high-dose interleukin-2 (IL-2), have been widely used. However, they benefit only a small subset of patients and are often associated with significant systemic toxicities^5,6^. Targeted therapies, including receptor tyrosine kinase inhibitors and immune checkpoint inhibitors, have significantly enhanced patient survival outcomes. However, their clinical application is challenged by issues such as the development of drug resistance to tyrosine kinase inhibitors^7^ and the absence of reliable predictive biomarkers for immune checkpoint inhibitors^8,9^. Consequently, there is an urgent need for more effective, biomarker-guided targeted therapies for the treatment of RCC.

von Hippel–Lindau (VHL) is a tumor suppressor gene (TSG) that exhibits the highest mutation frequency in RCC, occurring in over 50% of cases and serves as a key biomarker for the disease^10^. VHL encodes an E3 ubiquitin ligase that targets hypoxia-inducible factors (HIFs) for proteasomal degradation^11^. Loss of VHL function is strongly associated with RCC, which is characterized by aberrant angiogenesis and increased glucose dependence^12^, implicating HIF-mediated transcriptional reprogramming in tumor progression. Recent studies have revealed a strong link between VHL inactivation and genome-wide DNA hypermethylation in RCC. Robinson et al. demonstrated that VHL loss leads to substantial alterations in the DNA methylome, identifying 16,902 hypermethylated and 3932 hypomethylated CpG loci across three VHL-isogenic cell line pairs^13^. These findings suggest that VHL status exerts a global influence on DNA methylation, particularly promoting promoter hypermethylation across cell types. Further supporting this, Artemov and colleagues reported that RCC cells lacking functional VHL exhibit significantly increased DNA methylation, especially at promoter CpG islands^14,15^. Remarkably, reintroduction of VHL restored DNA methylation patterns to levels comparable to wild-type cells, underscoring VHL’s regulatory role in epigenetic remodeling in RCC.

DNA methylation is a key epigenetic regulatory mechanism involving the addition of methyl groups to cytosine residues in DNA, a process catalyzed by DNA methyltransferases (DNMT), such as DNMT1, DNMT3A and DNMT3B^16,17^. Ten–eleven translocation dioxygenases (TETs) are enzymes catalyzing DNA demethylation processes^18^. This modification plays a critical role in regulating gene expression^19^, maintaining genome stability and facilitating DNA repair^20,21^. Aberrant DNA methylation patterns, particularly the hypermethylation of TSGs such as *TP53* and *CDKN2A* (p16^INK4a^), have been widely implicated in tumor initiation and progression across various cancers^22–24^. In RCC, malignant transformation is accompanied by significant changes in the density and distribution of DNA methylation, with numerous genes undergoing epigenetic alterations^25,26^. Extensive research has demonstrated the diagnostic and therapeutic potential of DNA methylation signatures in RCC^27–31^. These findings underscore the critical role of DNA methylation in RCC pathogenesis and its promise as a biomarker for clinical applications.

In this study, we employed a synthetic lethal screening approach and identified that VHL-deficient RCC cells exhibit hyper-vulnerability to DNMT inhibitors. Both US Food and Drug Administration (FDA)-approved DNMT inhibitors, decitabine and azacitidine, as well as investigational small-molecule DNMT inhibitors, selectively suppressed the growth of VHL-deficient RCC cells. Mechanistic investigations revealed that this synthetic lethality is mediated through the reversal of hypermethylation at the promoter region of the putative TSG*KCNK3*. DNMT inhibition restored KCNK3 expression, leading to apoptosis in VHL-deficient RCC cells. These findings highlight DNA methylation as a promising therapeutic target and support the development of epigenetic-based strategies for treating patients with RCC with VHL loss.

## Materials and methods

### Cell lines and culture

The 786-O (RCC), H1975 (lung cancer), PLC/PRF/5 (liver cancer) and HEK293T cells were obtained from American Type Culture Collection (ATCC). 769-P (RCC) and Caki-1 (RCC) were obtained from the National Collection of Authenticated Cell Cultures. All the cell lines were authenticated by Short Tandem Repeat (STR) profiling (Applied Biosystems) and cultured according to provider’s instruction. 786-O, 769-P, H1975 and PLC/PRF/5 cell lines were maintained in RPMI 1640 with 10% fetal bovine serum (Thermo Fisher Scientific) and 1% penicillin–streptomycin. Caki-1 cells were maintained in MyCoy’s 5A medium with 10% fetal bovine serum (Thermo Fisher Scientific) and 1% penicillin–streptomycin. HEK293T cells were maintained in DMEM with 10% fetal bovine serum and 1% penicillin–streptomycin. All cells were cultured in 37 °C incubator with 5% CO_2_.

### Epigenetics compounds library screening

Epigenetics compounds library contains 138 small molecules which was purchased from Selleck Chemicals. Each compound was serially diluted at threefold concentration starting from a maximum concentration of 10 mM, resulting in a final eight gradient concentrations of each drug and added to 384-well plates before cell seeding. 786-O VHL-isogenic cell pair (786-O *VHL*^−/−^ cells and their counterparts overexpressing wild-type VHL, *wtVHL*^*OE*^ cells) were generated as previously described^32^ and seeded into two sets of diluted compounds libraries. After 3 days of incubation, cell viability and half-maximum inhibitory concentration (IC_50_) value was determined using GraphPad Prism software 9.3.1. The selection index (SI) value of each drug was calculated using the formula: SI = IC_50_ (786-O *wtVHL*^*OE*^)/IC50 (786-O *VHL*^−/−^), the candidate drugs were identified based on the SI ranking.

### Cell viability assay

Cell viability was detected with AlamarBlue solution which made by 0.025% (w/v) resazurin sodium salt (Sigma-Aldrich) dissolved in PBS. It was added to the cells with medium at a ratio of 10% and incubate for 3 h in 37 °C incubator with 5% CO_2_. The living cells would metabolize and converted the resazurin to fluorescent molecules, and the fluorescent signals were recorded by the SpectraMax M5 (Molecular Devices) at 560/590 nm (excitation/emission).

### Western blotting

Cells were lysed by the 2× Laemmli lysis buffer (62.5 mM Tris–HCl, pH 6.8, 10% glycerol, 1% SDS, 0.005% Bromophenol Blue, 10% 2-mercaptoethanol). Equivalent amounts of protein were loaded to 7.5%-12% SDS–polyacrylamide gel electrophoresis gels, transferred to a polyvinylidene fluoride membrane, blocked with 5% skim milk and immunoblotted with the specific primary antibodies at 4 °C overnight. On the following day, the membranes were incubated with the secondary antibody at room temperature for 2 h. After washing three times with PBST, images are captured by ChemiDoc MP Imaging System (Bio-Rad) with chemiluminescent substrate (Thermo Fisher Scientific). All antibodies used in this study are listed in Supplementary Table 2.

### siRNA transfection

All the siRNA were synthesized by Gene Universal (USA) and diluted to 20 μM with DEPC water. The siRNA transfection was conducted with lipofectamine RNA interference (RNAi) max reagent (Thermo Fisher Scientific). For high-throughput transfection, the reverse transfection was performed. In brief, siRNA and RNAi max reagent were added to Opti-MEM separately and mixed, then siRNA-lipid complexes were added into wells and incubated at room temperature for 5 min. Finally the cells and medium were added and cultured in 37 °C incubator with 5% CO_2_ for 48–72 h. Sequences of all siRNAs used in this study are listed in Supplementary Table 3.

### Annexin V/PI staining

Apoptotic cells were detected by Alexa Fluor 488 annexin V/Dead Cell Apoptosis Kit (Ivitrogen). Cells were collected, washed in cold PBS and resuspended in 1× annexin-binding buffer with a density of 1 × 10^6^ per milliliter. A total of 5 μL Alexa Fluor 488 annexin V and 1 μL 100 μg/mL PI working solution were added to each 100 μL of cell suspension. After incubation at room temperature for 15 min, the fluorescence of stained cells was detected by CytoFLEX flow cytometer (Beckman Coulter) at 530 nm and 575 nm using 488 nm excitation.

### Tumor xenograft mouse model

Female athymic nude mice (5–6 weeks old) were obtained and maintained by the Animal Research Core of University of Macau under specific pathogen-free conditions with controlled temperature, humidity and a 12-h light–dark cycle. Mice had ad libitum access to sterilized food and water. All animal procedures were approved by the Animal Research Ethics Committee of the University of Macau and conducted in accordance with institutional and international guidelines for animal care and use. To establish VHL-deficient RCC xenografts, VHL-deficient 786-O cells were collected and resuspended in prechilled PBS. An equal volume of Matrigel (Corning) was added to the cell suspension and mixed gently on ice to achieve a final concentration of 5 × 10^6^ cells per 100 μL. Each mouse was injected subcutaneously into the right flank using a 27-gauge needle. Tumor growth was monitored twice weekly by measuring the long (*L*) and short (*W*) axes with a digital caliper. Tumor volume was calculated using the ellipsoidal formula: tumor volume = π/6 × *L* × *W*^2^. When tumors became palpable (approximately 2 weeks post implantation), mice were randomized into treatment groups and administered either vehicle (PBS) or decitabine at 1 or 2 mg/kg via intraperitoneal injection twice weekly. Body weight was recorded at each measurement to monitor systemic toxicity. At the experimental endpoint (4 weeks after drug initiation), mice were euthanized by CO₂ asphyxiation. Tumors were excised, weighed and processed for downstream analyses. To evaluate the rescue effect of KCNK3 depletion on decitabine-induced synthetic lethality, mice were bilaterally injected with 1 × 10^7^ cells expressing either control short-hairpin RNA (shCTRL) or shKCNK3 (100 μL per site, mixed with Matrigel as above). Tumor growth was slower in shRNA-expressing xenografts; therefore, tumor size and body weight were measured every 2 weeks. Mice were administered either PBS or decitabine at 2 mg/kg via intraperitoneal injection twice weekly. Treatment continued until the experimental endpoint (10 weeks after drug initiation). Mice were killed as described above, and tumors were collected for weight measurement and further molecular analyses.

### Generation of patient with RCC-derived cell model

RCC tissue specimens were obtained from patients at Renji Hospital. All patients were pathologically confirmed to have RCC, as detailed in Supplementary Table 1. The study protocol was approved by the Ethics Committee of Renji Hospital (Shanghai, China, approval no. RA-2023-237). Before sample collection, informed consent was obtained from all participants. All experimental procedures were conducted in accordance with the ethical guidelines authorized by the Ethics Committee of Renji Hospital. Fresh RCC tissue specimens were obtained from patients via surgical excision or biopsy (Supplementary Table 1). Necrotic regions were excluded, and the remaining tissue was finely minced into 2–3-mm fragments using sterile scissors. The fragments were enzymatically digested in primary medium containing 0.15% collagenase type I (Sigma-Aldrich, C0130), 0.1% dispase (Gibco, 17105-041) and 0.04% hyaluronidase (Sigma-Aldrich, H3506) at 37 °C for 30–180 min. After digestion, the tissue was aspirated using a pipette tip, gently dispersed as much as possible and collected into a 15 mL centrifuge tube. The single-cell suspension is then adjusted to 10–15 mL with RPMI 1640. The cell suspension centrifuged at 1000 rpm for 5 min. The culture dishes were precoated with rat tail collagen type I (Corning, 354236) at a concentration of 5 μg/cm². The pelleted cells were resuspended in primary medium and seeded onto collagen type I-coated culture dishes at a desired cell density (for example, 1 × 10^5^ cells for a 6 cm dish). The culture medium was prepared by RPMI 1640 medium (Gibco) supplemented with 10% fetal bovine serum, 40 ng/mL epidermal growth factor (EGF, Gibco, PHG0313), 1% insulin–transferrin–selenium–sodium pyruvate (Gibco, 51300044) and 10 μM Y-27632 (DC Chemicals, DC45762). Cultures were maintained in a humidified incubator at 37 °C with 5% CO₂ until epithelial cell clones emerged. These clones were transferred to new culture dishes for expansion and proliferation. Once the cells reached 80–90% confluency, they were enzymatically detached using 0.05% trypsin-EDTA (Gibco, 25300054) and passaged at a ratio of 1:3. The culture medium was refreshed every 2–3 days. All cells were maintained in the incubator at 37 °C with 5% CO₂.

### VHL mutational characterization of patient with RCC-derived cells

To characterize the mutation type of VHL in patients with RCC-derived cells, whole-exome sequencing was performed and analyzed. For quality control, FastQC (v0.11.9) was used to assess the raw sequencing data. Trimmomatic (v0.39) was employed to remove adapter sequences and low-quality reads. Specifically, adapter sequences and leading N bases with a quality score below 3 were trimmed. A sliding window approach (window size of four bases) was applied to trim reads when the average quality per base fell below 15 and removing those shorter than 30 bases. The cleaned data were aligned to the human reference genome (UCSC_hg38) using BWA MEM (v0.7.17-r1188). Samtools v1.6 (using htslib 1.6) was utilized to convert SAM format files to BAM format, followed by sorting and indexing. Duplicate reads were initially marked using the MarkDuplicates tool from GATK (v4.3.0.0) and subsequently removed with Picard (v2.27.5). Base quality score recalibration (BQSR) was performed using the BaseRecalibrator and ApplyBQSR tools in GATK. To identify somatic mutations in tumor samples, we used Mutect2 for SNP and indel calling, following best practices. Germline variants were filtered out using established databases including dbSNP146, the 1000 Genomes Project and genomAD. Variants retained for downstream analysis were those annotated in the COSMIC database, indicating potential somatic origin. Indels were further filtered using the Mills_and_1000G_gold_standard resource. Due to the absence of matched normal tissues in the available patient with RCC-derived cell models, we employed a commonly used workflow to call probable somatic mutations in cancer cell model analysis. Finally, the results were annotated using ANNOVAR.

### RT–qPCR

All the RNA samples were extracted from cells with RNeasy Plus Mini Kit (Qiagen), the concentration of RNA was determined by Nanodrop 2000 (Thermo Scientific), then 2 μg of RNA of each sample was used for reverse transcription via a high-capacity cDNA reverse transcription kit (Thermo Fisher Scientific) to obtain cDNA. Taq Universal SYBR Green Supermix (Bio-Rad) was used to preparation of reaction mixture that is loaded into CFX96 Real-Time PCR System (Bio-Rad) to quantitative PCR analysis. The sequence information of all the reverse transcription and quantitative real-time PCR (RT–qPCR) primers used in this study is shown in Supplementary Table 4.

### RNA-sequencing

Total RNA samples were extracted using the TRIzol reagent (Invitrogen) After library construction, Illumina Novaseq 5000 was used to perform paired-end sequencing. The sequences were processed by fastp software to obtain the clean data with high quality. Reference genome (GRCh38) was downloaded from the UCSC website, then reads were mapped to the human reference genome via HISAT2 (v2.2.0). Quantification of gene expression level was calculated by featureCounts v1.5.0-p3. DESeq2 package was used to differential expression analysis and the adjusted *P* value of 0.05 was set as the threshold for significantly differential expression. Gene set enrichment analysis (GSEA) was conducted using the clusterProfiler R package and visualized with GSEAplot2.

### Bisulfite sequencing PCR

Genomic DNA were extracted by PureLink Genomic DNA Mini Kit (K1820-00, Invitrogen). Then, bisulfite conversion was conducted according to the instruction of EpiTect Fast DNA Bisulfite Kit (69824, Qiagen). Primers were designed using Methyl Primer Express v1.0 software. The target DNA fragment was amplified by 2× EpiArt HS Taq Master Mix through Nested PCR. Then, the purified PCR products were cloned into T-vector with pClone007 Versatile Simple Vector Kit (Tsingke). After transformation, ten colonies were picked per sample for sequencing. The results were analyzed by BiQ Analyzer software. The bisulfite sequencing PCR primers of KCNK3 used in this study are listed in Supplementary Table 5.

### Methylation-specific PCR

Extracted genomic DNA was bisulfite converted by using EZ DNA Methylation-Gold Kit (Zymo Research). Two sets of primers were designed via MethPrimer (https://www.methprimer.com/) for determining the methylated and unmethylated DNA. Then bisulfite-treated genomic DNA was subjected to PCR amplification with ZymoTaq DNA Polymerase (Zymo Research). The PCR products were loaded on to 1.5% agarose gel and electrophoresis for 30 min at 80 V. The methylation-specific PCR primers of KCNK3 used in this study are listed in Supplementary Table 6.

### Plasmids and transfection

The 786-O and HEK293T cells were seeded in six-well plates 1 day in advance. Then solution A was prepared by diluting 3.75 μL Lipofectamine 3000 reagent (Thermo Fisher Scientific) in 125 μL Opti-MEM medium (Thermo Fisher Scientific), solution B was prepared by diluting 5 μg KCNK3 (GeneCopoeia) plasmid in 125 μL Opti-MEM medium and subsequently added 10 μL P3000 reagent. Solution B was added into solution A and incubate 10 min at room temperature. The DNA-lipid complex was dripped into six-well plates with adherent cells. After incubation cells for 2–4 days at 37 °C, the cell viability was detected with AlamarBlue assay and images were taken with EVOS (Invitrogen, Thermo Fisher Scientific).

### Construction of KCNK3-knockdown cell with shRNA

HEK293T cells were cultured in a 10 cm dish at a density of 70%. Then KCNK3 lentiviral shRNA vector (GeneCopoeia), pCMV-dR8.2 dvpr and pCMV-VSV-G (9 μg:10 μg:1 μg) were transfected into HEK293T cells with lipo3000. After 16 h transfection, the cell medium was replaced with new medium containing 30% serum and the cells were cultured for an additional 48 h. The shKCNK3 lentiviral particles was collected by centrifugation of cell culture medium at 1,500 rpm for 5 min and passed through a 0.45 μm filter. The 786-O cells were seeded in six-well plates at a density of 70%. On the following day, 1 mL shKCNK3 lentiviral particles and 5 μg/mL polybrene were added into medium. After incubation for 48 h, transduced cells were selected with 2 μg/mL puromycin for 3 days and seeded into 96-well plates at a concentration of one cell per well to obtain single colony cells with KCNK3 knockdown.

### Statistical methods

All experiments were repeated at least three times. Statistical analyses were performed using GraphPad Prism software 9.3.1. Statistical significances between two groups were analyzed by Student’s *t*-test. Statistical significances between two curves were analyzed by two-way analysis of variance (ANOVA). The *P* value <0.05 was considered to be statistically significant.

## Results

### Identification of DNMT inhibitors as synthetic lethal drugs for VHL-deficient RCC

**Fig. 1:**
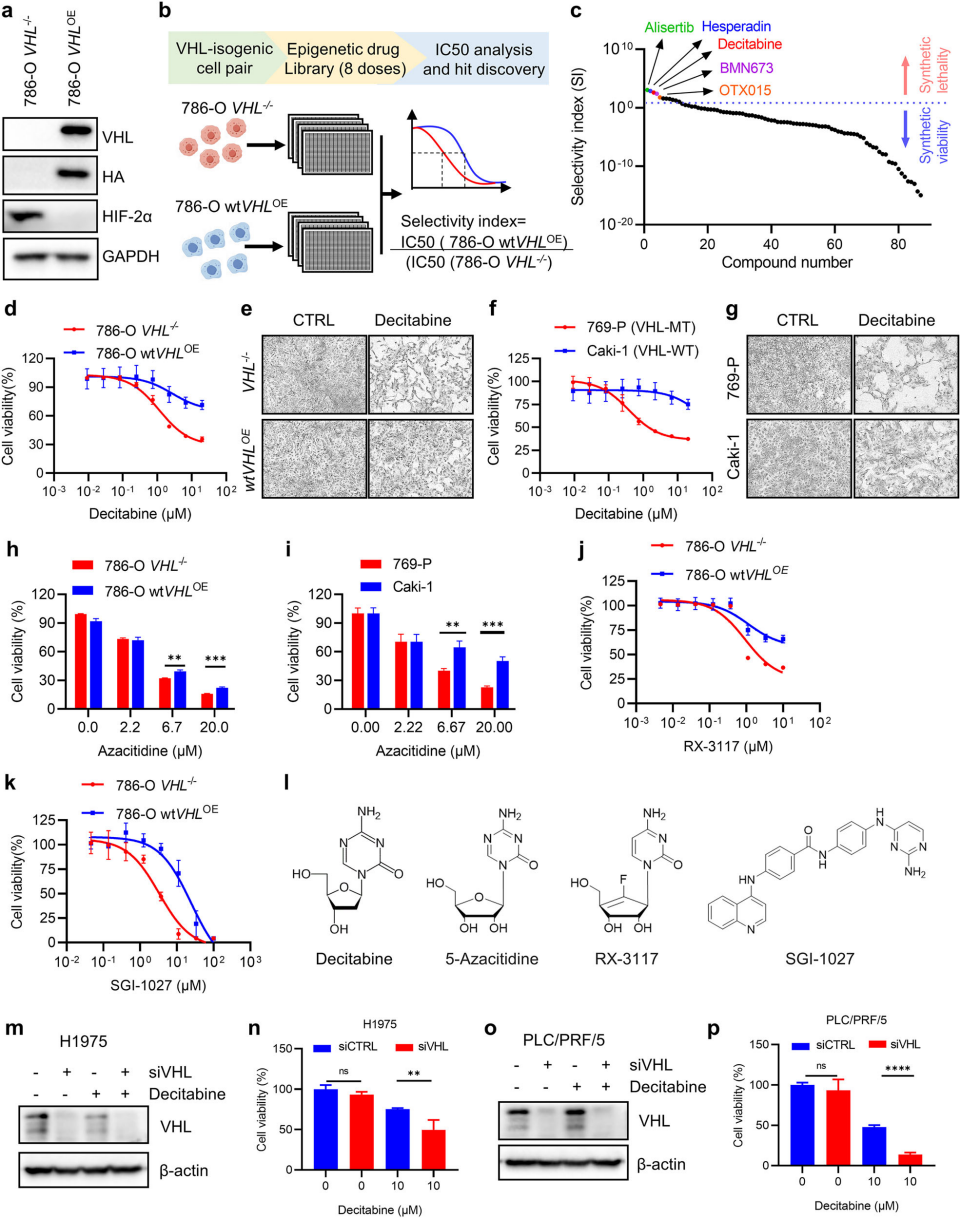
Identification of DNMT inhibitors as synthetic lethal drugs for VHL-deficient RCC. **a** Stable overexpression of VHL-HA plasmid in 786-O cells (a VHL mutated RCC cell). Western blotting detected the protein level of VHL, HA tag and its downstream target genes HIF-2α in this VHL-isogenic 786-O cell pair. Data are shown as the mean ± s.d., *n* = 3. **b** A schematic diagram of synthetic lethal drug screen using VHL-isogenic 786-O cell pair on the Epigenetics Compound Library. **c** Selectivity index (fold-difference in IC_50_ values between *VHL*^−/−^ and *wtVHL*^*OE*^ cells) plot to identify top five synthetic lethal drug candidates. **d–g** Dose–response curve of 786-O VHL-isogenic cell pair (**d**) and VHL non-isogenic cell pair (**f**) treated with decitabine for 72 h. Data are shown as the mean ± s.d., *n* = 3. The representative images of cell density with the indicated concentration of decitabine for *wtVHL*^*OE*^ and *VHL*^−/−^ (**e**) and Caki-1 and 769-p (**g**) cells. **h**,**i** Azacitidine effect on cell viability in 786-O VHL-isogenic cell pair (**h**) and VHL non-isogenic cell pair (**i**). Data are shown as the mean ± s.d., *n* = 3. **P < 0.01, ***P < 0.001 between two indicated groups, Student’s *t*-test. **j**,**k** Dose–response curve of 786-O VHL-isogenic cell pair treated with RC-3117 (**j**) and SGI-1027 (**k**). Data are shown as the mean ± s.d., *n* = 3. **l** Chemical structures of DNMT inhibitors used in this study. **m–p** Western blotting detected the protein level of VHL in H1975 (**m**) and PLC/PRF/5 cells (**o**) and decitabine effect on cell viability of VHL-depleted H1975 cells (**n**) and PLC/PRF/5 (**p**). Data are shown as the mean ± s.d., *n* = 3. **P < 0.01, ****P < 0.001 between two indicated groups, Student’s *t*-test.

To identify therapeutic vulnerabilities in VHL-deficient RCC, we conducted a synthetic lethal target screen using a VHL-isogenic RCC cell pair—786-O cells lacking functional VHL (*VHL*^−/−^) and their counterparts overexpressing wild-type VHL (*wtVHL*^*OE*^) (Fig. 1a). This screen utilized a target-defined, human epigenetic compound library comprising 128 small molecules targeting diverse epigenetic regulators. Conducted in 384-well plates using an eight-dose interplate titration format, the screen generated IC₅₀ values for each compound across both cell lines. Compounds exhibiting selective hypersensitivity in *VHL*^−/−^ cells compared with *wtVHL*^*OE*^ cells were identified as synthetic lethal candidates (Fig. 1b). The top five hits included aurora kinase inhibitors alisertib and hesperadin, the DNMT inhibitor decitabine, the PARP inhibitor BMN673 (talazoparib) and the BET inhibitor OTX015 (birabresib) (Fig. 1c). Given that synthetic lethality of aurora kinase inhibitors in VHL-deficient cancers has been previously reported^33,34^, we prioritized decitabine—the second highest-ranking hit—for further investigation. Decitabine is an FDA-approved drug indicated to use in the treatment of myelodysplastic syndromes, including chronic myelomonocytic leukemia, and is actively investigated for other indications^35,36^. We subsequently validated the screening results using both VHL-isogenic and non-isogenic RCC cell lines, along with additional DNMT inhibitors. Notably, decitabine selectively inhibited the proliferation of VHL-deficient 786-O and 769-P cells, while having a reduced effect on 786-O cells overexpressing wild-type VHL (786-O *wtVHL*^*OE*^) and Caki-1 cells, which naturally express wild-type VHL (Fig. 1d–g). Similarly, the FDA-approved DNMT inhibitor azacitidine, as well as investigational agents RX-3117^37^ and SGI-1027^38^, also preferentially suppressed the growth of VHL-deficient RCC cells (Fig. 1h–k). Structurally, decitabine, azacitidine and RX-3117 are cytosine-based nucleoside analogs, whereas SGI-1027 is a non-nucleoside DNMT inhibitor (Fig. 1l), suggesting that a broad spectrum of DNMT inhibitors can induce synthetic lethality in VHL-deficient RCC. Furthermore, VHL-depleted non-RCC cancer cell lines, including the lung cancer cell line H1975 and the hepatocellular carcinoma cell line PLC/PRF/5, also exhibited increased sensitivity to decitabine (Fig. 1m–p). These findings indicate that the synthetic lethal interaction between VHL loss and DNMT inhibition is conserved across multiple cancer types.

### DNMT inhibition is synthetic lethal in VHL-deficient RCC in vivo

**Fig. 2:**
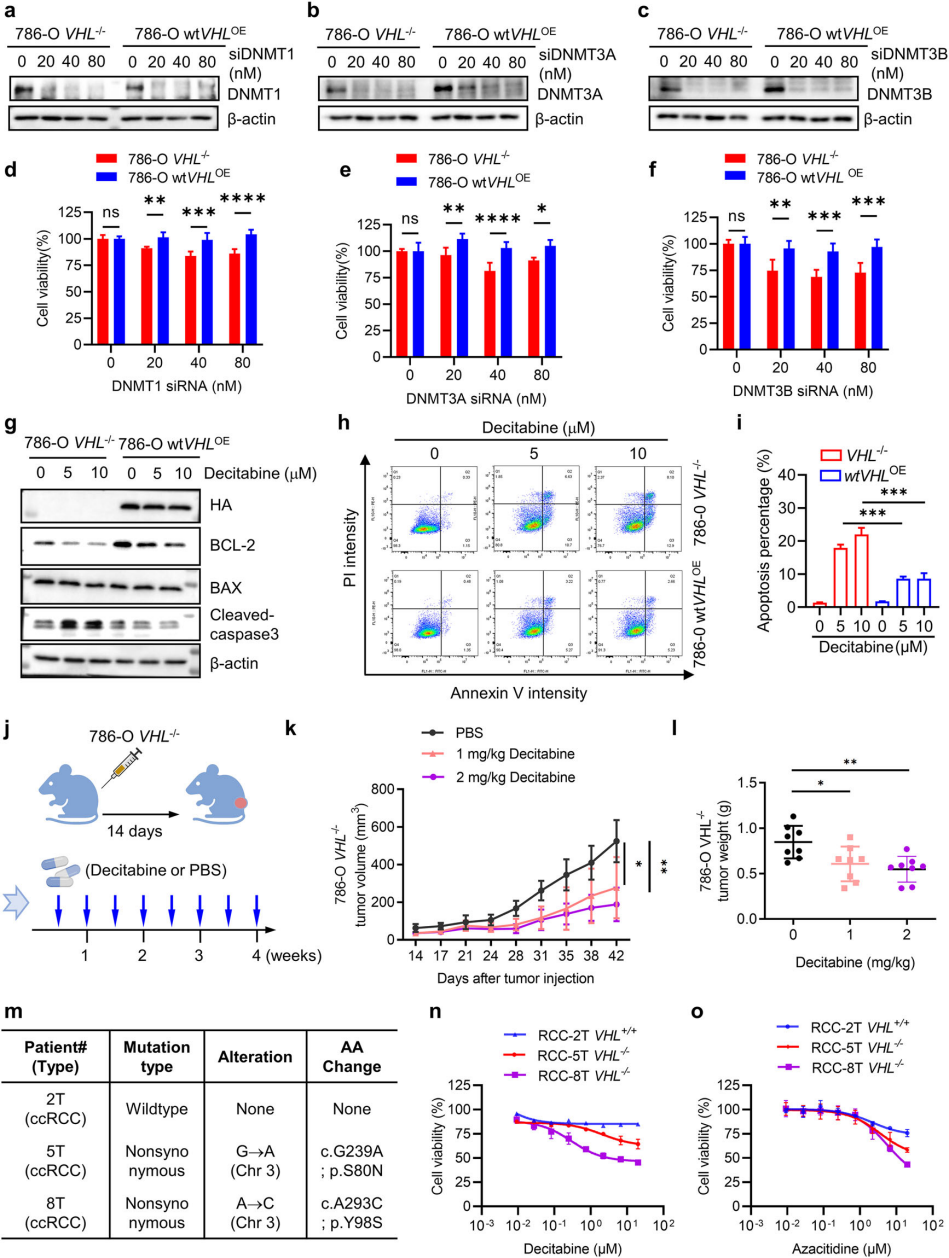
DNMT inhibition is synthetic lethal in VHL-deficient RCC in vivo. **a**–**f** Validation of the synthetic lethality between DNMT and VHL using siRNA depletion of individual DNMT isoforms. Knockdown efficiency of each DNMT isoform following siRNA transfection is shown in panels **a**–**c**. Synthetic lethal effects resulting from the depletion of individual DNMT isoforms are presented in panels **d**–**f**. Data are shown as the mean ± s.d., *n* = 3. *P < 0.05, **P < 0.01, ****P < 0.001, ****P < 0.0001 between two indicated groups, Student’s *t*-test. **g**–**i** Decitabine effect on apoptosis in 786-O VHL-isogenic cell pairs; 786-O VHL-isogenic cell lines were treated with indicated concentration of Decitabine for 72 h, the protein level of HA, BCL-2, BAX and cleaved-caspase-3 were detected by western blotting (**g**). The apoptotic cells were detected by flow cytometry with Alexa Fluor 488 annexin V/PI staining (**h**) and performed statistical analysis of apoptotic cells (**i**). **j** A schematic diagram of the mouse xenograft experiments. The 786-O cells were implanted subcutaneously, and 14 days later, mice received intraperitoneal injections with PBS or decitabine (1 and 2 mg/kg) twice per week for a duration of 4 weeks. **k**–l Decitabine inhibited tumor growth in the 786-O xenograft mice model, with the tumor growth curve (**k**) and tumor wet weight (**l**) analysis of 786-O tumor xenograft mice treated with or without decitabine. Data are shown as the mean ± s.d., *n* = 8, *P < 0.05, **P < 0.01 between two indicated groups, two-way ANOVA. **m**–**o** Validation of synthetic lethality between DNMT and VHL in PDC; mutational characterization of VHL in the RCC PDC (**m**) and dose–response of decitabine (**n**) and azacitidine (**o**) on patients with RCC-derived cells.

To determine whether the synthetic lethal effects of DNMT inhibitors in VHL-deficient RCC cells are specifically attributable to DNMT inhibition rather than off-target drug effects, we individually silenced DNMT isoforms—DNMT1, DNMT3A and DNMT3B—using siRNAs and assessed cell viability in VHL-isogenic RCC cell pairs. Silencing each DNMT isoform partially recapitulated the synthetic lethality observed in VHL-deficient cells (Fig. 2a–f). These partial effects probably reflect the distinct and nonredundant roles of DNMT isoforms in DNA methylation: DNMT1 functions as a maintenance methyltransferase, whereas DNMT3A and DNMT3B mediate de novo methylation. Although decitabine and azacitidine primarily target DNMT1, they are also known to inhibit DNMT3A and DNMT3B. Consistent with this, decitabine treatment in our study led to measurable reductions in the protein levels of all three DNMT isoforms (Supplementary Fig. 1), suggesting that the synthetic lethal effects are probably due to combined inhibition of multiple DNMTs. To further investigate the cellular consequences of DNMT inhibition, we analyzed cell death markers following decitabine treatment. In 786-O *VHL*^−/−^ cells, decitabine markedly increased cleaved caspase-3 levels and reduced BCL-2 expression (Fig. 2g). Annexin V/PI staining confirmed that decitabine preferentially induced apoptosis in VHL-deficient cells (Fig. 2h,i). These findings indicate that DNMT inhibitor-induced synthetic lethality in VHL-deficient RCC is mediated by apoptosis.

To validate these findings in vivo, we performed RCC xenograft experiments in athymic nude mice bearing 786-O *VHL*^−/−^ tumors. Mice were treated with PBS (vehicle control) or decitabine at 1 or 2 mg/kg twice weekly for 4 weeks (Fig. 2j). Tumor growth was monitored by measuring tumor volume and wet weight. Decitabine significantly suppressed tumor growth at both doses (Fig. 2k,l). Importantly, body weights of treated and control mice remained stable and comparable throughout the treatment period (Supplementary Fig. 2), indicating that decitabine was well tolerated and did not induce overt toxicity at this dosage regimen. To more robustly validate the synthetic lethal effect of DNMT inhibitors in VHL-deficient RCC, we collected tumor samples from patients with RCC at Renji Hospital and established patient-derived cancer cell (PDC) lines. Three PDC lines—RCC-2T, RCC-5T and RCC-8T—were successfully cultured. Whole-exome sequencing was performed to characterize VHL mutations in these lines. The analysis revealed that RCC-2T harbors wild-type VHL, whereas RCC-5T (S80N) and RCC-8T (Y98S) carry pathogenic VHL mutations (Fig. 2m). We subsequently assessed the sensitivity of these PDCs to DNMT inhibitors. Drug response data demonstrated that VHL-mutant PDCs were consistently more sensitive to DNMT inhibitors, including decitabine and azacitidine, compared to the VHL wild-type PDC (Fig. 2n,o). Collectively, these results demonstrate that DNMT inhibition induces synthetic lethality in VHL-deficient RCC cells through apoptosis and highlight DNMT inhibitors as strong candidates for VHL-specific antitumor agents.

### VHL deficiency upregulates DNMT1 expression via HIF-2α

**Fig. 3:**
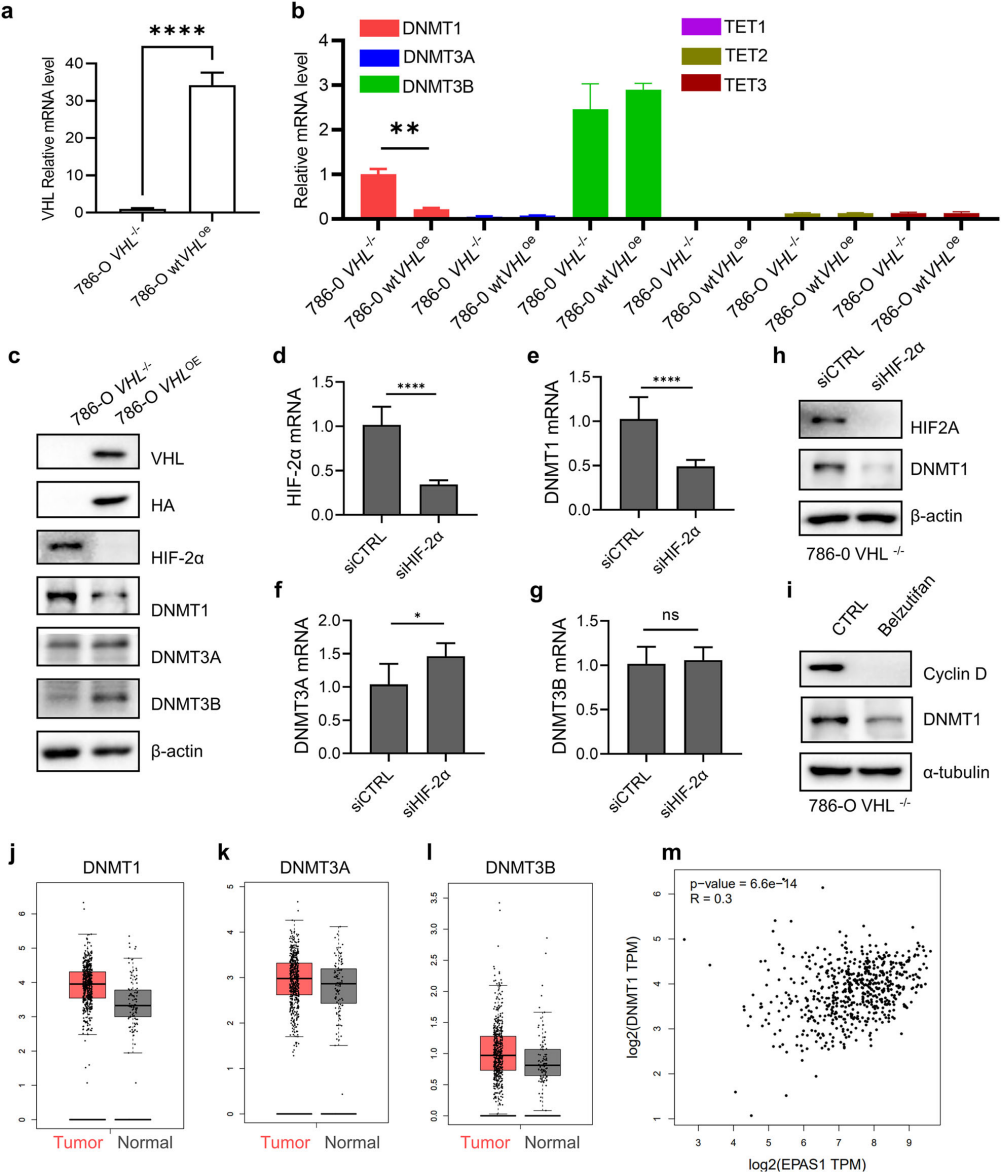
VHL deficiency upregulates DNMT1 expression via HIF-2α. **a**,**b** mRNA levels of VHL, DNMT isoforms and TET isoforms in 786-O VHL-isogenic cell pair. Data are shown as the mean ± s.d., *n* = 6, **P < 0.01, ****P < 0.0001 between two indicated group, Student’s *t*-test. **c** Western blotting analysis showed the protein level of VHL, HA, HIF-2α, DNMT1, DNMT3A and DNMT3B in 786-O VHL-isogenic cell pair. **d**–**g** Effects of HIF-2α siRNA on the mRNA levels of HIF-2α (**d**) and DNMT1 (**e**), DNMT3A (**f**) and DNMT3B (**g**). Data are shown as the mean ± s.d., *n* = 6, *P < 0.05, ****P < 0.0001 between two indicated group, Student’s *t*-test. **h**,**i** Effect of HIF-2α siRNA (**h**) or HIF-2α inhibitor belzutifan (**i**) on the protein level of DNMT1. Cyclin D was used as a positive control for HIF-2α target gene expression. **j**–**l** Clinical data analysis of DNMT1 (**j**), DNMT3A (**k**) and DNMT3B (**l**) in kidney tumor and normal tissue samples. The box plots showed the expression of DNMT1, DNMT3A and DNMT3B by GEPIA tool (http://gepia.cancer-pku.cn/index.html). **m** Correlation analysis of DNMT1 and EPAS1 (HIF-2α) transcript levels in patients with kidney cancer tumor samples by GEPIA tool.

The observed synthetic lethality between VHL loss and DNMT inhibition suggests a potential role for VHL in regulating DNA methylation in RCC cells. Previous studies have reported that VHL-deficient RCC cells exhibit genome-wide CpG hypermethylation^13–15,39^. These studies raised the possibility that VHL may influence the expression of DNA methylation enzymes such as DNMTs or TETs. To explore this hypothesis, we examined the impact of VHL status on the expression of DNMT and TET family members in RCC cells. Our results revealed that DNMT1 messenger RNA levels were significantly elevated in VHL-deficient RCC cells compared with their VHL wild-type counterparts (Fig. 3a,b). By contrast, the expression levels of DNMT3A, DNMT3B and TET enzymes did not show significant differences between the two groups. Western blot analysis further confirmed that DNMT1 protein levels were upregulated in VHL-deficient cells (Fig. 3c). To determine whether this upregulation is mediated by HIF-2α, we depleted HIF-2α in VHL-deficient RCC cells and assessed DNMT1 expression. Depletion of HIF-2α led to a significant reduction in DNMT1 mRNA (Fig. 3d–g) and protein levels (Fig. 3h). Treatment with belzutifan, a small-molecule HIF-2α inhibitor^40^, similarly decreased DNMT1 expression (Fig. 3i), indicating that DNMT1 upregulation in VHL-deficient RCC is driven by HIF-2α activation. Further supporting this mechanism, DNMT1 expression was found to be higher in kidney tumor tissues compared with normal kidney tissues (Fig. 3j–l). Moreover, transcriptomic analysis of patient samples revealed a positive correlation between DNMT1 and EPAS1 (encoding HIF-2α) expression in kidney cancer (Fig. 3m). Together, these findings demonstrate that VHL deficiency leads to HIF-2α activation, which in turn upregulates DNMT1 expression, contributing to CpG hypermethylation in RCC cells. This mechanistic link provides further insight into the basis of synthetic lethality between VHL loss and DNMT inhibition.

### Identification of TSGs that mediate the synthetic lethal effects of DNMT inhibitors

**Fig. 4:**
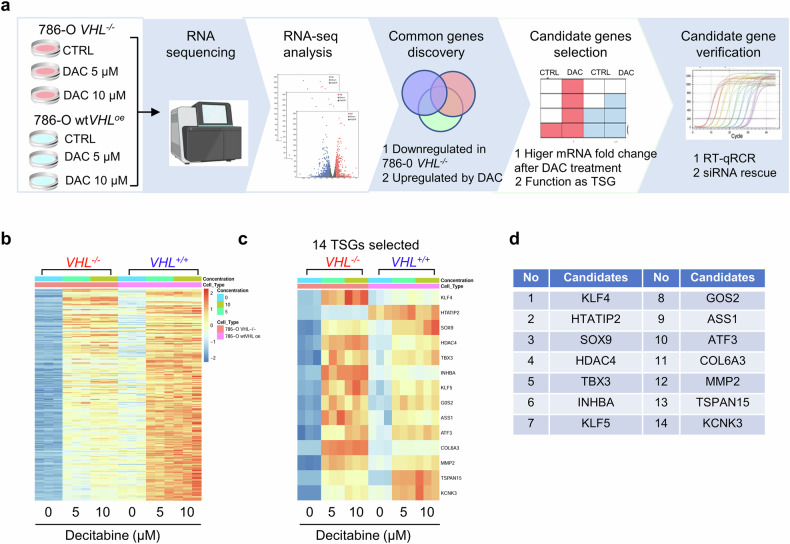
Identification of TSGs that mediate the synthetic lethal effects of DNMT inhibitors. **a** A schematic illustration of RNA-sequencing and TSG identification using VHL-isogenic 786-O cell pair treated with or without decitabine (DAC). **b** A heat map shows the enrichment and transcription of DEGs that are downregulated in VHL-deficient cells compared with VHL wild-type cells and are upregulated by DAC treatment. **c**,**d** Identification of candidate TSGs that are repressed in VHL-deficient cells and are derepressed by DAC treatment. A heat map depicting selected TSGs that are downregulated in VHL‑deficient cells relative to VHL wild‑type cells and upregulated upon DAC treatment is shown in panel **c**. The corresponding list of the 14 selected TSGs is presented in panel **d**.

Alterations in the CpG methylation landscape of tumors frequently involve hypermethylation of promoter regions encompassing the first exon in TSGs, resulting in their epigenetic silencing^41^. We hypothesized that specific TSGs are epigenetically silenced in VHL-deficient RCC cells due to promoter CpG methylation, and treatment with DNMT inhibitors could restore their expression. This reactivation may inhibit cell growth by overwhelming the cells with tumor-suppressive signals. To test this hypothesis and identify TSGs that contribute to the synthetic lethal effects of DNMT inhibition, we performed transcriptome profiling on VHL-isogenic RCC cell pairs treated with two concentrations (5 and 10 μM) of decitabine (Fig. 4a). We focused on differentially expressed genes (DEGs) that were downregulated in VHL-deficient cells relative to VHL wild-type cells and were upregulated following decitabine treatment (Supplementary Fig. 3a–e). A total of 398 DEGs met these criteria across both decitabine doses (Fig. 4b and Supplementary Fig. 3f). These DEGs were further screened for known or putative TSG functions using the TSGene database (https://bioinfo.uth.edu/TSGene/index.html) and OncoK database (https://www.oncokb.org/), and the ranking was based on the difference in log_2_ fold change after decitabine treatment between VHL-deficient cells and VHL wild-type cells (Fig. 4c). This analysis identified 14 candidate TSGs, including KLF4, INHBA, COL6A3, ASS1 and KCNK3, whose expression was substantially suppressed in VHL-deficient RCC cells and restored by decitabine treatment (Fig. 4c,d). These genes represent potential mediators of the synthetic lethality induced by DNMT inhibitors.

### KCNK3 is hypermethylated in VHL-deficient RCC and derepressed by DNMT inhibition

**Fig. 5:**
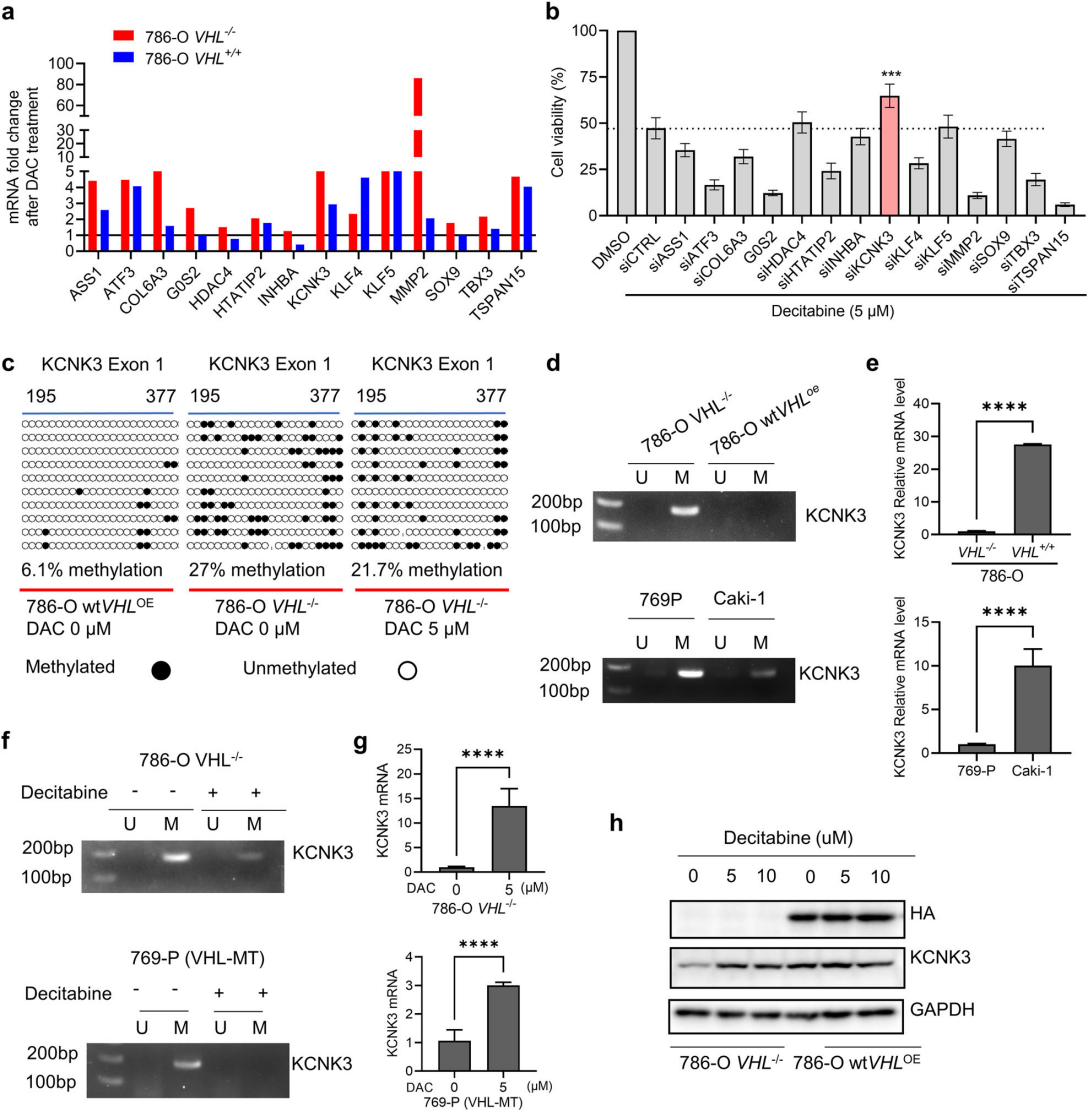
Identification of KCNK3 is hypermethylated in VHL-deficient RCC and derepressed by DNMT inhibition. **a** RT–qPCR validation of the 14 TSG mRNA expression between 786-O *VHL*^−/−^ and wt*VHL*^OE^ cells treated with decitabine (DAC). **b** siRNA rescue screening for 14 candidate genes in 786-O *VHL*^−/−^ cells treated with DAC. Data are shown as the mean ± s.d., *n* = 3, ***P < 0.001 between two indicated group, Student’s *t*-test. **c** Bisulfite methylation sequencing in 786-O VHL-isogenic cell pair treated with or without DAC is shown. **d** Agarose gel electrophoresis analyses of methylation-specific PCR products for KNCK3 in indicated cells are shown. **e** RT-qPCR analyses measuring KCNK3 mRNA levels in indicated cells are presented. **f** Agarose gel electrophoresis analyses of methylation-specific PCR products for KNCK3 in cells treated with or without DAC are shown. **g** RT-qPCR analyses measuring KCNK3 mRNA levels in cells treated with or without DAC are presented. Data are shown as the mean ± s.d., *n* = 3, ****P < 0.0001 between two indicated group, Student’s *t*-test. **h** Western blotting analysis showed the protein level of KCNK3 in 786-O VHL-isogenic cell pair treated with or without DAC.

To investigate the regulation of 14 candidate TSGs in VHL-isogenic cells, we treated cells with decitabine and performed an RNAi rescue screen to identify TSGs mediating synthetic lethality. Most of the 14 TSGs were markedly upregulated upon decitabine treatment, particularly in VHL-deficient cells (Fig. 5a). We designed siRNAs targeting each of the 14 TSGs and assessed their ability to rescue VHL-deficient RCC cells from decitabine-induced growth inhibition. Among these, only KCNK3 silencing significantly rescued cell viability (Fig. 5b). KCNK3 (also known as TASK-1) encodes a potassium channel and has been reported as a tumor suppressor whose downregulation correlates with poor prognosis in various cancers^42^. To determine whether KCNK3 is a direct DNMT target contributing to synthetic lethality, we examined its methylation status. MethPrimer (https://www.methprimer.com/) analysis predicted CpG islands in the promoter and first exon regions of KCNK3 (Supplementary Fig. 4). Bisulfite sequencing revealed hypermethylation of the first exon in 786-O *VHL*^−/−^ cells compared with 786-O *wtVHL*^OE^ cells, which was reversed by decitabine treatment (Fig. 5c). Methylation-specific PCR confirmed increased methylation in VHL-deficient cells (Fig. 5d), correlating with reduced KCNK3 mRNA levels (Fig. 5e). Decitabine treatment significantly reduced methylation and restored KCNK3 transcription (Fig. 5f,g). Western blot analysis showed higher KCNK3 protein levels in *wtVHL*^OE^ cells than in *VHL*^−/−^ cells, and decitabine increased KCNK3 protein expression only in VHL-deficient cells (Fig. 5h). These findings demonstrate that KCNK3 is epigenetically silenced via exon 1 hypermethylation in VHL-deficient RCC cells, and DNMT inhibition reverses this silencing, restoring KCNK3 expression. Since we observed that HIF-2α regulates DNMT1 transcription and affects gene hypermethylation in VHL-deficient RCC cells, we investigated the role of HIF-2α in regulating DNMT1 and KCNK3 gene methylation. We overexpressed wild-type HIF-2α in VHL wild-type RCC (ACHN) cells, where endogenous HIF-2α levels are nearly undetectable. Successful overexpression was confirmed by western blot and RT–qPCR (Supplementary Fig. 5a–d). As expected, DNMT1 mRNA and protein levels increased substantially upon HIF-2α overexpression (Supplementary Fig. 5a–b). However, HIF-2α overexpression did not promote KCNK3 gene methylation or suppress its transcription (Supplementary Fig. 5c–e). By contrast, silencing HIF-2α in VHL-deficient RCC cells reduced DNMT1 mRNA and markedly increased KCNK3 expression, accompanied by decreased KCNK3 methylation (Supplementary Fig. 5f–j). These findings suggest that HIF-2α-mediated DNMT1 transcription is necessary but not sufficient for KCNK3 methylation and transcriptional repression. KCNK3 methylation probably requires DNMT1 in combination with other factors, such as TET activity suppression, under the condition of VHL loss or hypoxia.

### KCNK3 mediates DNMT inhibitor-induced synthetic lethality in VHL-deficient RCC

**Fig. 6:**
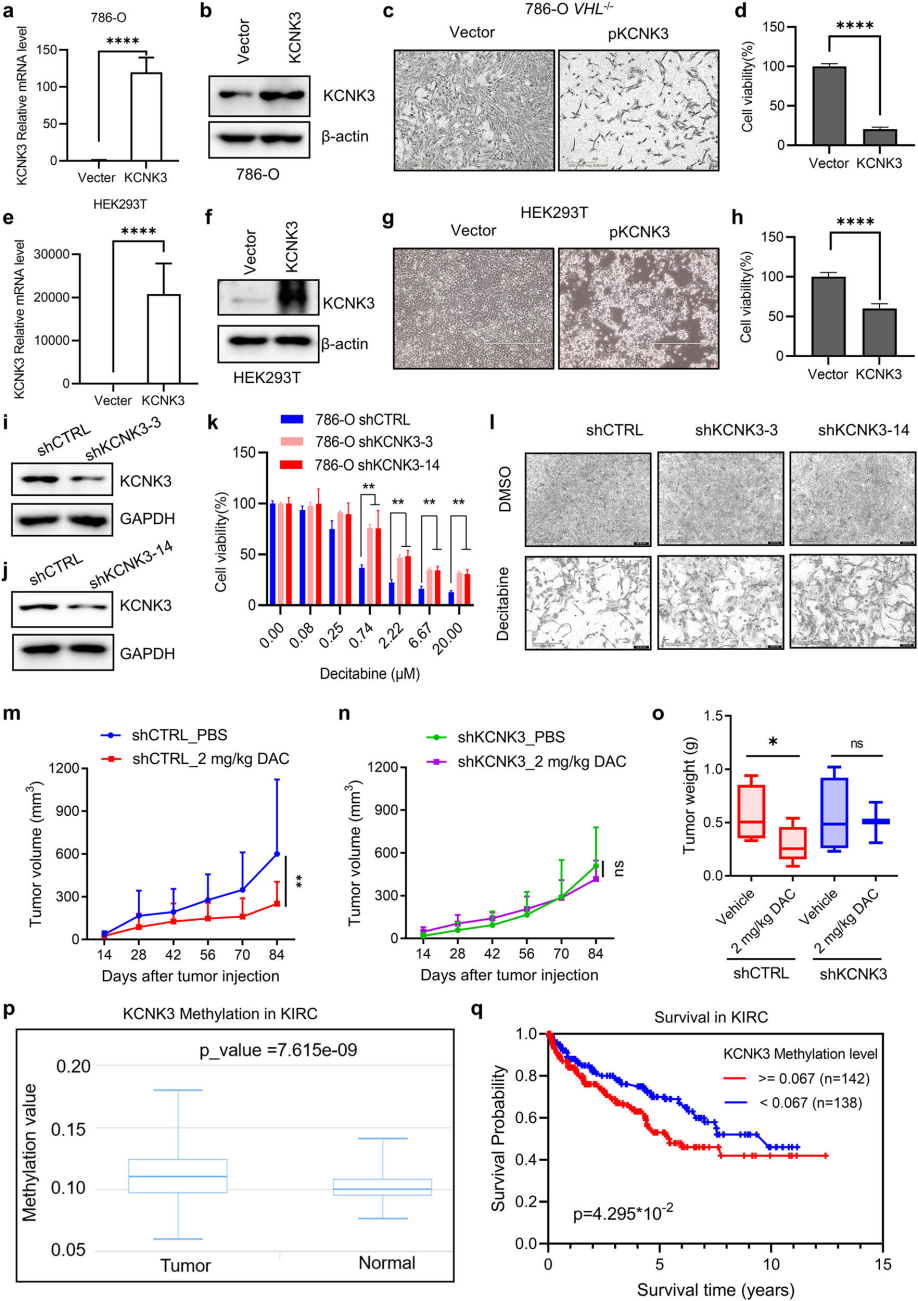
KCNK3 mediates DNMT inhibitor-induced synthetic lethality in VHL-deficient RCC. **a–h** The effect of KCNK3 ectopic overexpression on cell viability in 786-O *VHL*^−/−^ cells (**a**–**d**) and HEK293T cells (**e**–**h**). Verification of KCNK3 overexpression in 786-O *VHL*^−/−^ cells by RT-qPCR (**a**) and Western blotting (**b**) is shown. The effects of KCNK3 overexpression on 786-O *VHL*^−/−^ cell viability are presented in panels **c** and **d**, including representative cell images (**c**) and quantitation of viability (**d**). Verification of KCNK3 overexpression in HEK293 cells by RT-qPCR (**e**) and Western blotting (**f**) is shown. The effects of KCNK3 overexpression on HEK293 cell viability are shown in panels **g** and **h**, with representative cell images (**g**) and quantitation (**h**). Data are shown as the mean ± s.d., *n* = 3, ****P < 0.0001 between two indicated group, Student’s *t*-test. **i-l** Rescue effects of KCNK3 depletion (shKCNK3) on the synthetic lethality induced by decitabine (DAC) in vitro. shControl and two shKCNK3 clones (shKCNK3-3 (**i**) and shKCNK3-14 (**j**)) in 786-O cells were tested. Western blotting analysis showed protein level of KCNK3 in **i** and **j**; 786-O shCTRL cells and shKCNK3 cells were treated with DAC for 72 h. **k** The cell viability was detected by alarm blue assay. **l** The representative images of cell density of 786-O shCTRL cells and shKCNK3 cells treated with indicated DAC were taken by IncuCyte Zoom. **m**–**o** Rescue effects of KCNK3 depletion (shKCNK3) on the synthetic lethality induced by DAC in mouse xenograft experiments. Tumor growth curve for shCTRL cells (**m**) and shKCNK3 cells (**n**), tumor wet weight (**o**) analysis of 786-O shCTRL or shKCNK3 cell xenograft mice treated with PBS or DAC. Data are shown as the mean ± s.d., *n* = 5, *P < 0.05, **P < 0.01 between two indicated groups, two-way ANOVA. **p** Clinical data analysis of KCNK3 gene methylation level in kidney renal clear cell carcinoma (KIRC) tumor versus normal tissues. The data originate from the DiseaseMeth version 2.0 (http://bio-bigdata.hrbmu.edu.cn/diseasemeth/index.html). **q** Patient with KIRC survival analysis between KCNK3 gene high methylation and low methylation cohorts. The data originate from EWAS (https://ngdc.cncb.ac.cn/ewas/datahub/index).

To validate KCNK3 as a mediator of synthetic lethality, we examined the effects of its genetic perturbation. Ectopic overexpression of KCNK3 in 786-O and HEK293 cells significantly suppressed cell proliferation (Fig. 6a–h), supporting its tumor suppressor role. Conversely, shRNA-mediated knockdown of KCNK3 in VHL-deficient RCC cells rescued the synthetic lethal effects of decitabine (Fig. 6i–l). In xenograft models, decitabine (2 mg/kg) suppressed tumor growth in 786-O cells expressing shCTRL but not in cells expressing shKCNK3 (Fig. 6m–o). These results confirm that KCNK3 contributes to DNMT inhibitor-induced synthetic lethality in VHL-deficient RCC. Clinical data analysis revealed significantly higher KCNK3 methylation in RCC tumors compared with normal tissues (Fig. 6p), and elevated methylation was associated with poor patient survival (Fig. 6q). Thus, KCNK3 methylation is a prognostic marker in RCC and a critical DNMT inhibitor target mediating synthetic lethality.

### KCNK3 promotes TNF-α production, MAPK pathway activation and pro-apoptotic process in decitabine-treated VHL-deficient RCC cells

**Fig. 7:**
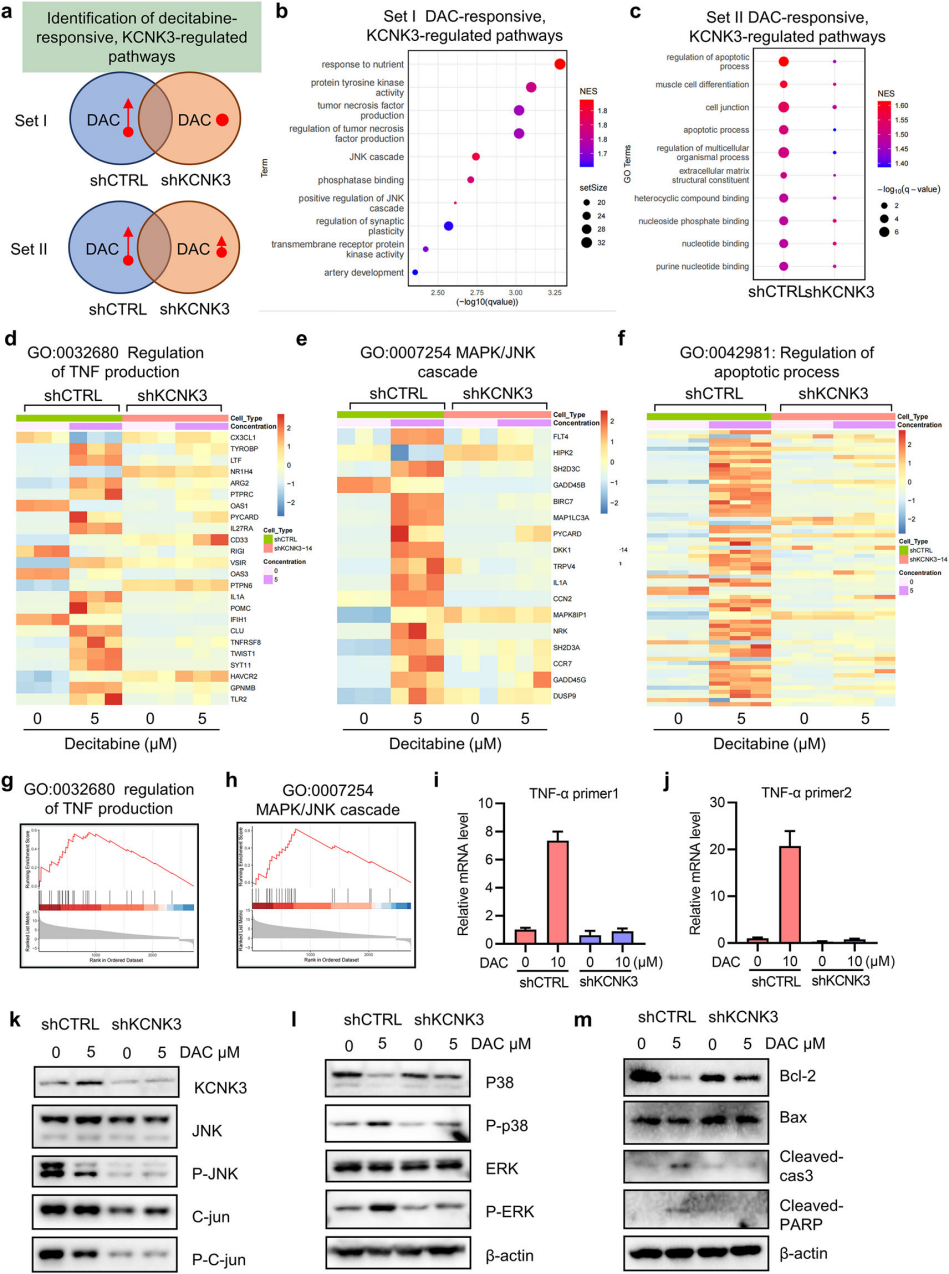
KCNK3 promotes TNF-α production, MAKP pathway activation, and pro-apoptotic process in DAC-treated VHL-deficient RCC cells. **a** RNA-sequencing analysis of transcriptome profiles in 786-O shCTRL and shKCNK3 cells treated with or without decitabine (DAC). A Venn diagram shows the two sets of KCNK3-regulated, DAC effector pathways. Set Ι: a GSEA shows the upregulated pathway in shCTRL cells treated with DAC but not in shKCNK3 cells. Set ΙΙ: a GSEA shows the upregulated pathway in shCTRL cells treated with DAC with higher NES score. **b** Top ten pathways involved in set Ι. **c** Top ten pathways involved in set ΙΙ. **d**–**f** A heat map shows the mRNA level of genes involved in the regulation of tumor necrosis factor production (**d**), MAPK/JNK cascade (**e**) and regulation of apoptotic process (**f**) pathways. **g**,**h** GSEA enrichment plots showed the regulation of tumor necrosis factor production (**g**) and MAPK/JNK cascade (**h**) pathways. **i**,**j** KCNK3 depletion reversed the DAC induced-TNF-α production. 786-O shCTRL cells and shKCNK3 cells treated with or without DAC, RT–qPCR analysis of mRNA level of TNF-α with two different pairs of primers (TNF-α primer 1 (**i**) and TNF-α primer 2 (**j**)). **k**–**m** KCNK3 depletion effect on MAKP pathway and apoptosis pathway. 786‑O shCTRL and shKCNK3 cells were treated with or without DAC, and Western blot analyses were performed to assess the protein levels of KCNK3 and key components of the MAPK pathway, including JNK/C‑Jun (**k**) and p38 and ERK (**l**), as well as proteins involved in the apoptosis pathway (**m**).

**Fig. 8:**
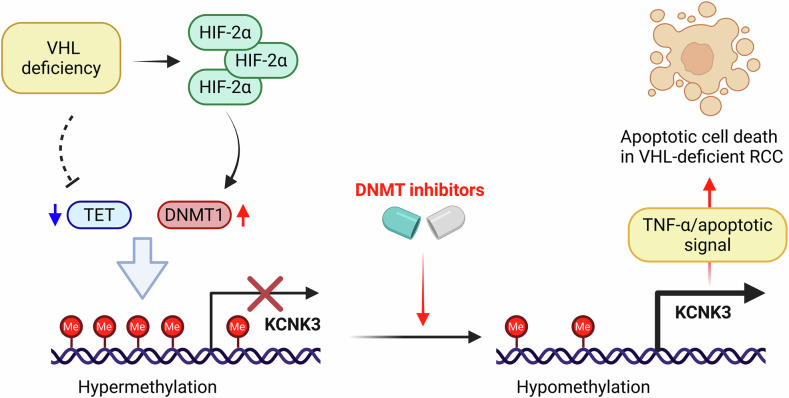
Mechanism model of synthetic lethality between DNMT inhibition and VHL loss. In VHL wild-type cells, HIF-2α is degraded, resulting in lower DNMT1 expression. Consequently, TSGs remain hypomethylated and are expressed normally, leading to minimal response to decitabine treatment. In VHL-deficient cells, HIF-2α is accumulated and DNMT1 transcription is elevated. Independently of HIF-2α, VHL deficiency and hypoxic condition could also suppress the activity of TETs, oxygen-dependent dioxygenase enzymes. These combined events could induce hypermethylation and repression of certain TSGs. Among these candidate TSGs, KCNK3 emerges as a key mediator of decitabine-induced synthetic lethality. In VHL-deficient cells, KCNK3 undergoes CpG hypermethylation and transcriptional silencing, which can be reversed by decitabine treatment. The upregulation of KCNK3 subsequently activates TNF-α and apoptotic signaling, contributing to synthetic lethality in VHL-deficient RCC. Figure created with BioRender.com (Shim, J. (2025)).

Although KCNK3 overexpression mediates synthetic lethality in VHL-deficient RCC cells, its downstream mechanisms remain unclear. To elucidate these, we performed RNA-sequencing on 786-O shCTRL and shKCNK3 cells treated with or without decitabine. GSEA revealed two distinct categories of gene sets (set I and set II) in response to decitabine treatment. Set I comprised gene sets that were significantly upregulated in shCTRL cells but remained unchanged in shKCNK3 cells. Set II comprised gene sets that were strongly upregulated in shCTRL cells, yet only marginally induced in shKCNK3 cells (Fig. 7a). Set I included genes involved in nutrient response, TNF production and MAPK/JNK signaling, whereas set II was enriched for apoptotic processes (Fig. 7b–h). We were particularly interested in TNF, MAPK/JNK and apoptotic process because these pathways are functionally interconnected. Canonical TNF-α signaling (TNF-R1 pathway) can elicit two distinct cellular outcomes: prosurvival and pro-apoptotic responses. These divergent fates are mediated through downstream activation of the MAPK/JNK signaling cascade and the apoptotic machinery^43^. Transcript analysis showed that TNF-α expression was strongly induced by decitabine in shCTRL cells, but this induction was abolished in shKCNK3 cells (Fig. 7i,j). Other TNF-related genes followed a similar pattern (Supplementary Fig. 6a). Western blot analysis revealed that JNK phosphorylation decreased, whereas p38 and ERK phosphorylation increased in shCTRL cells following decitabine treatment (Fig. 7k,l). These effects were absent in shKCNK3 cells, suggesting that KCNK3 overexpression by decitabine activates p38 and ERK pathways. In addition, Bcl-2 levels decreased, and cleaved caspase-3 and PARP increased in shCTRL cells treated with decitabine, indicating apoptosis induction. These pro-apoptotic effects were diminished in shKCNK3 cells (Fig. 7m and Supplementary Fig. 6b), indicating that the pro-apoptotic phenotypes of decitabine is primarily mediated by KCNK3 overexpression. Together, these findings suggest that VHL loss in RCC induces KCNK3 gene hypermethylation and epigenetic silencing, and DNMT inhibition reverses the KCNK3 silencing, promoting TNF-α-mediated apoptotic cell death in VHL-deficient RCC cells (summarized in Fig. 8).

## Discussion

In this study, we identified DNMT inhibitors as potent synthetic lethal agents for VHL-deficient RCC. Through both in vitro and in vivo analyses, we demonstrated that DNMT inhibition selectively induces apoptosis in VHL-deficient RCC cells, offering a promising therapeutic strategy for this genetically defined RCC subtype. Notably, structurally diverse DNMT inhibitors elicited similar synthetic lethal effects, which were also observed in cancer types beyond RCC, suggesting broad applicability of this approach. Our xenograft experiments confirmed the efficacy and tolerability of decitabine, an approved DNMT inhibitor, in suppressing tumor growth in VHL-deficient RCC models. Corroborating our experimental findings, analysis of a patient with RCC data revealed a positive correlation between DNMT1 and HIF-2α expression, elevated methylation of the KCNK3 gene in tumor versus normal tissues, and an association between high KCNK3 methylation and poor patient survival. These findings underscore the potential of DNMT inhibitors as VHL-specific antitumor agents.

Mechanistically, we found that VHL loss leads to upregulation of DNMT1 via HIF-2α activation, suggesting a direct link between VHL deficiency and aberrant DNA methylation. Previous studies have reported CpG hypermethylation in VHL-deficient RCC and other cancers, yet the underlying mechanisms remain incompletely understood^13–15,39^. Thienpont and coworkers demonstrated that tumor hypoxia causes DNA hypermethylation by reducing TET activity^44^. The authors that TET catalytic activity is reduced in various types of cells under hypoxia and this reduction is independent of hypoxia-associated alterations in TET expression. They further showed that TET is an oxygen-dependent enzyme that catalyzes DNA demethylation through 5-methylcytosine oxidation and the oxygen shortage under hypoxia condition reduces the TET activity, thereby increasing hypermethylation at gene promoters. In addition to the change in TET activity under hypoxia, several literatures suggested the presence of hypoxia-responsive elements (HREs) in the promoters of DNMTs^45,46^. Xu et al. demonstrated that there are at least seven putative HRE sites presented in the promoter region of DNMT1 in non-small cell lung cancer cells^46^. HIF-2α was accumulated under hypoxic condition and this in turn transactivated DNMT1 expression in non-small cell lung cancer, suggesting HIF-2α binding to HREs as a regulatory mechanism of DNMT1 transcription. Consistent with this, our JASPAR analysis (https://jaspar.elixir.no/) predicted one high-confidence HRE (≥80% profile score) and 32 additional sites (≥70%) within the DNMT1 promoter (data not shown). In RCC cells, we observed that HIF-2α activation in VHL-deficient RCC promotes DNMT1 expression. Both genetic silencing and pharmacological inhibition of HIF-2α reduced DNMT1 levels. Conversely, DNMT1 mRNA and protein levels increased significantly upon HIF-2α overexpression. Furthermore, DNMT1 expression positively correlated with HIF-2α levels in patients with RCC tumors, suggesting that HIF-2α contributes to DNA hypermethylation in VHL-deficient cells by upregulating DNMT1. However, HIF-independent mechanisms—such as hypoxia-induced TET inhibition—may also play a role in DNA hypermethylation. Although our HIF-2α overexpression increased DNMT1 expression level, it did not promote KCNK3 gene methylation or suppress its transcription. By contrast, silencing HIF-2α in VHL-deficient RCC reduced DNMT1 mRNA and increased KCNK3 expression, accompanied by decreased KCNK3 gene methylation. These findings suggest that HIF-2α-mediated DNMT1 transcription is necessary but not sufficient for KCNK3 methylation. Together, it can be postulated that VHL loss and hypoxic conditions suppress TET activity, and together with DNMT1 elevation by HIF-2α, contribute to KCNK3 gene hypermethylation. Our results indicate that HIF-2α–DNMT1 signaling plays an indispensable role in maintaining DNA hypermethylation.

The epigenetic dysregulation in VHL-deficient RCC cells appears to silence a subset of TSGs, which can be reactivated by DNMT inhibition. Transcriptomic profiling identified 14 candidate TSGs whose expression was suppressed in VHL-deficient cells and restored upon decitabine treatment, supporting the hypothesis that DNMT inhibition exerts its synthetic lethal effect by reactivating silenced TSGs. Among these, KCNK3 was identified as a key mediator of the synthetic lethality based on two main considerations: (1) among the 14 TSGs derepressed by decitabine, our RNAi rescue experiments demonstrated that only KCNK3 knockdown significantly reversed decitabine-induced effects, indicating its unique functional role in this synthetic lethal pathway. Although other TSGs were also derepressed by decitabine, their silencing did not rescue the phenotype, suggesting they may play supportive rather than central roles. (2) Mechanistically, KCNK3 encodes a potassium channel that facilitates efflux of intracellular potassium ions. Intracellular potassium homeostasis is critical for multiple cellular processes, including cell survival and apoptosis. For example, elevated intracellular potassium is known to inhibit caspase cascade and suppress apoptosis^47^. In this regards, KCNK3 activation could lower intracellular potassium level, potentially relieving caspase inhibition and promoting apoptotic cell death. We found that KCNK3 is hypermethylated at its first exon in VHL-deficient RCC cells, leading to transcriptional silencing. Decitabine treatment reversed this hypermethylation, restoring both mRNA and protein expression of KCNK3. Functional assays confirmed its tumor suppressor role: ectopic expression of KCNK3 suppressed cell proliferation, whereas its knockdown rescued cells from decitabine-induced apoptosis. In vivo, KCNK3 knockdown abrogated the antitumor effects of decitabine, further validating its role in mediating synthetic lethality. Importantly, clinical data showed that KCNK3 methylation is significantly elevated in RCC tumors and correlates with poor patient survival, positioning KCNK3 as both a prognostic biomarker and a potential therapeutic target.

KCNK3 is a member of the potassium channel protein family, which regulates potassium ion flow to maintain cellular physiological functions^48^. It plays diverse roles in cellular processes, including neuronal activity, T cell activation, hypoxia sensing, glucose tolerance and mitochondrial function^49–52^. Dysregulation of KCNK3 has been implicated in several diseases, such as pulmonary arterial hypertension, atrial fibrillation and sleep apnea^53,54^, highlighting its clinical relevance. Recent studies suggest that KCNK family genes may serve as prognostic markers in breast and liver cancers^55,56^, and their expression is frequently altered in malignancies^57^, indicating a potential role in tumorigenesis. Specifically, KCNK3 has been shown to suppress proliferation and metabolism in non-small cell lung cancer, with its tumor suppressor function linked to the AMPK–TXNIP pathway and glucose metabolism. Despite these insights, the mechanisms by which KCNK3 inhibits RCC remain poorly understood. To address this, we performed transcriptomic profiling to identify decitabine-responsive pathways regulated by KCNK3. Our analysis revealed significant enrichment of TNF-α signaling, MAPK/JNK pathways and apoptotic signaling. These pathways were substantially upregulated in control cells treated with decitabine but were blunted in KCNK3-depleted cells. Western blot analyses confirmed that decitabine-induced KCNK3 expression leads to reduced Bcl-2 levels and increased cleaved caspase-3 and PARP, hallmark indicators of apoptosis. Our findings align with previous reports that canonical TNF-α signaling can lead to both prosurvival and pro-apoptotic outcomes, depending on the cellular context and downstream signaling dynamics^58^. Our data suggest that KCNK3 overexpression induces TNF-α-mediated apoptosis in VHL-deficient RCC cells, whereas MAPK activation may represent a compensatory survival response. Together, these findings support a model in which KCNK3 mediates synthetic lethality through modulation of apoptotic and stress-response pathways in RCC.

In summary, this study identifies KCNK3 as a key epigenetically silenced TSG in VHL-deficient RCC and demonstrates that its reactivation by DNMT inhibitors mediates synthetic lethality through pro-apoptotic signaling. These insights provide a compelling rationale for the clinical evaluation of DNMT inhibitors in VHL-deficient RCC and highlight the potential of KCNK3 methylation as a biomarker for patient stratification.

## Supplementary information

Supplementary Information
