# Supplementary material for: DNA methyltransferase inhibition is a therapeutic vulnerability in VHL-deficient renal cell carcinoma cells
Source: Exp Mol Med. 2026 Mar 6;58(3):798–812. doi: 10.1038/s12276-026-01663-w (PMC13049176; doi:10.1038/s12276-026-01663-w)
Supplement: Supplementary file 1 — Supplementary Information [file 12276_2026_1663_MOESM1_ESM.pdf]

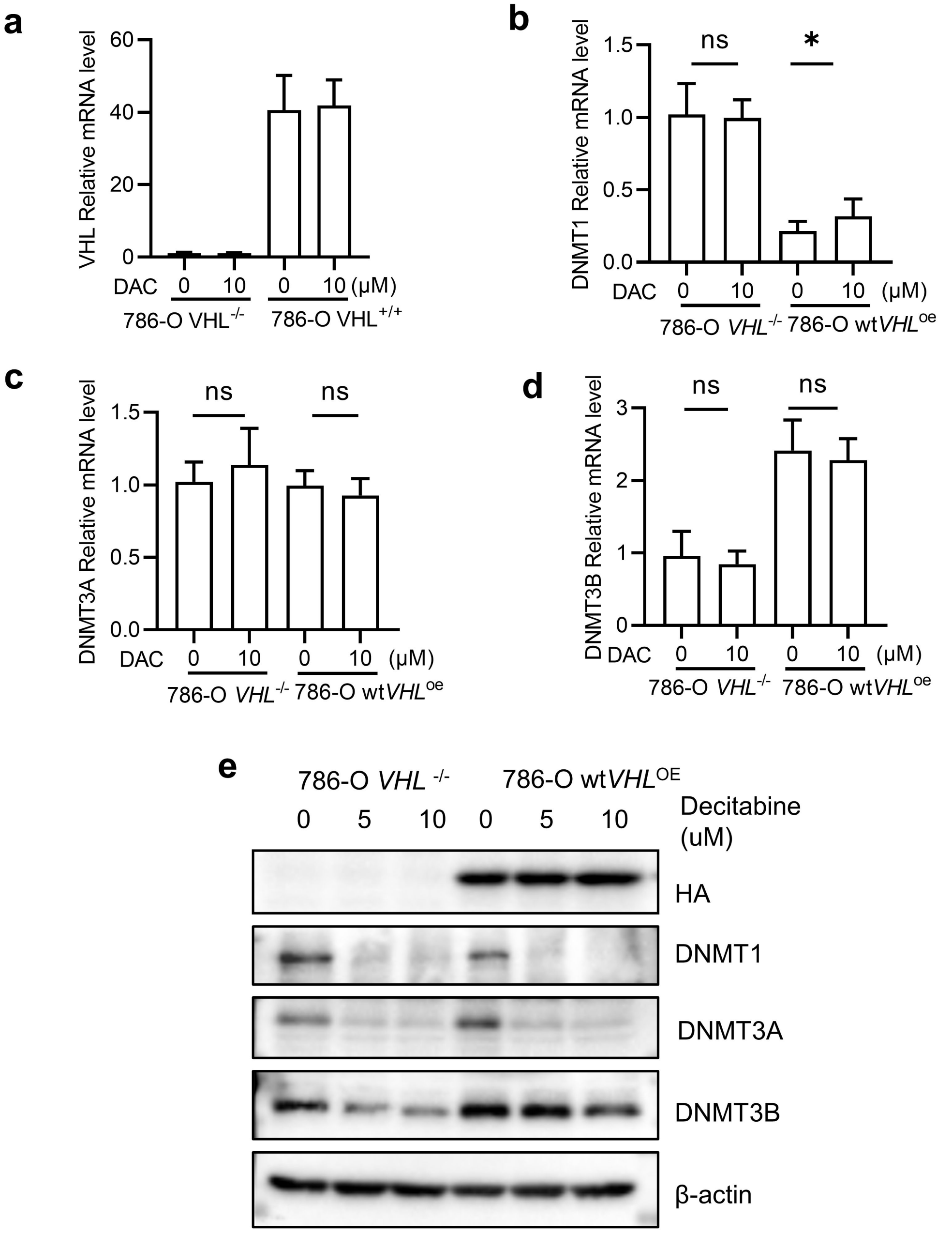

Supplementary Figure 1 Decitabine effect on the mRNA level and protein level of DNMT1, DNMT3A and DNMT3B. VHL-isogenic 786-O cell lines were treated cell with 10 μM Decitabine for 48 hours. RT-qPCR analysis of VHL (**a**), DNMT1 (**b**), DNMT3A (**c**) and DNMT3B (**d**) mRNA level. Western blotting analysis of HA, DNMT1, DNMT3A and DNMT3B protein level (**e**).

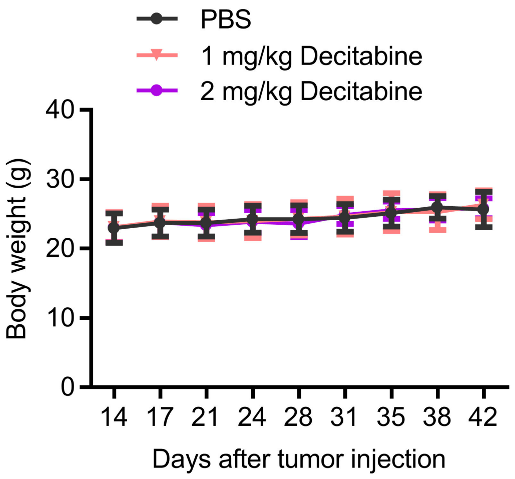

Supplementary Figure 2 Decitabine effect on 786-O xenograft mice model. Body weight analysis of 786-O tumor xenograft mice treated with or without decitabine. Data are shown as the mean ± SD, n=8.

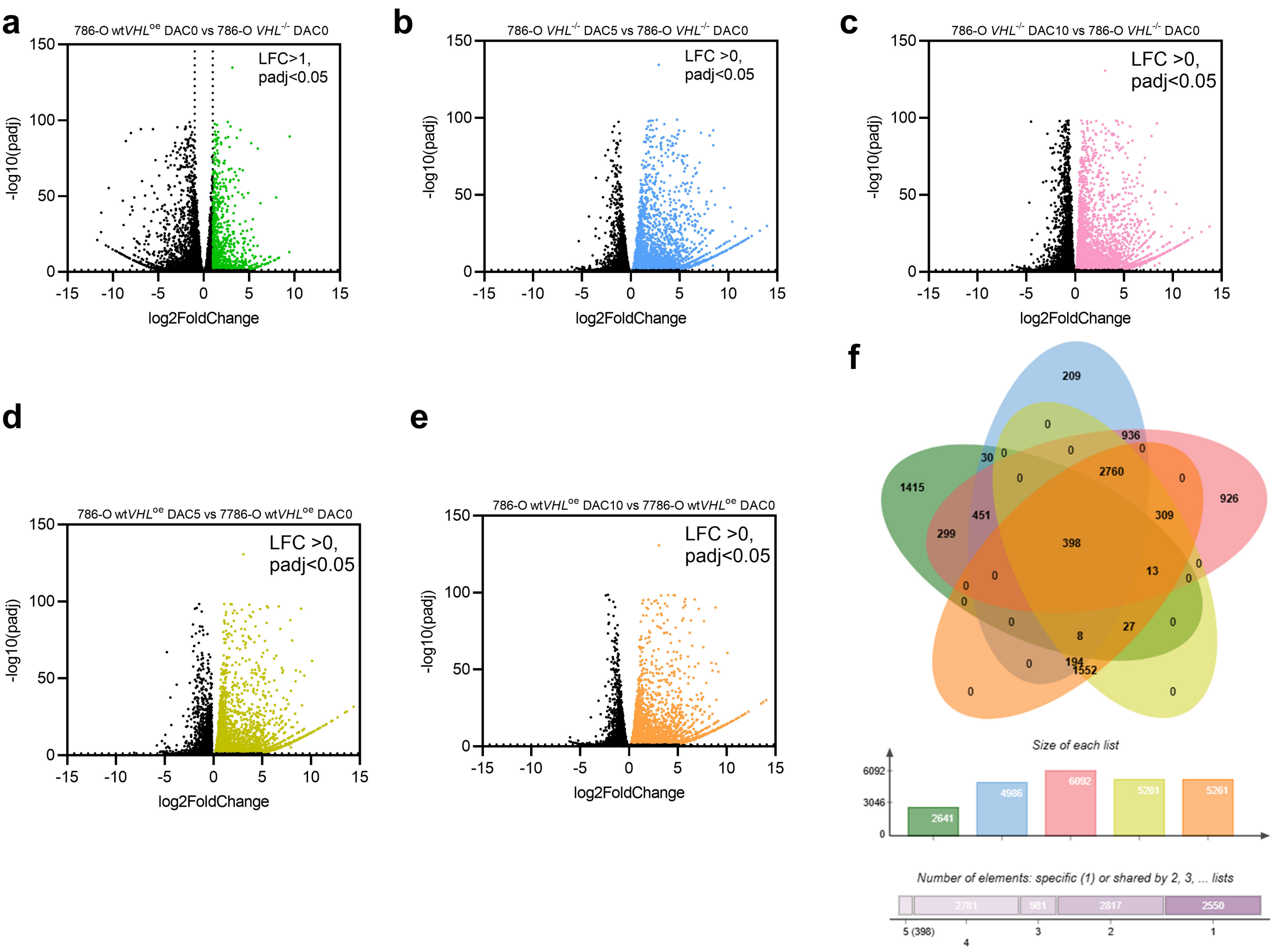

Supplementary Figure 3 Volcano plots of DEGs in each experimental condition. The down regulated genes in 786-0 *VHL*$^{-/-}$ cells compared with 786-O wt*VHL*^{OE} cells, log2FC>1, padj<0.05 (**a**). The up regulated genes in 786-O *VHL*$^{-/-}$ cells treated with 5 μM (**b**) or 10 μM (**c**) Decitabine (DAC) for 48 hours, log2FC>0, padj<0.05. The up regulated genes in 786-O wt*VHL*^{OE} cells treated with 5 μM (**d**) or 10 μM (**e**) Decitabine (DAC) for 48 hours, log2FC>0, padj<0.05. Venn diagram (**f**) showed enrichment of DEGs that are downregulated in VHL-deficient cells compared to VHL-wildtype cells and are upregulated by decitabine treatment.

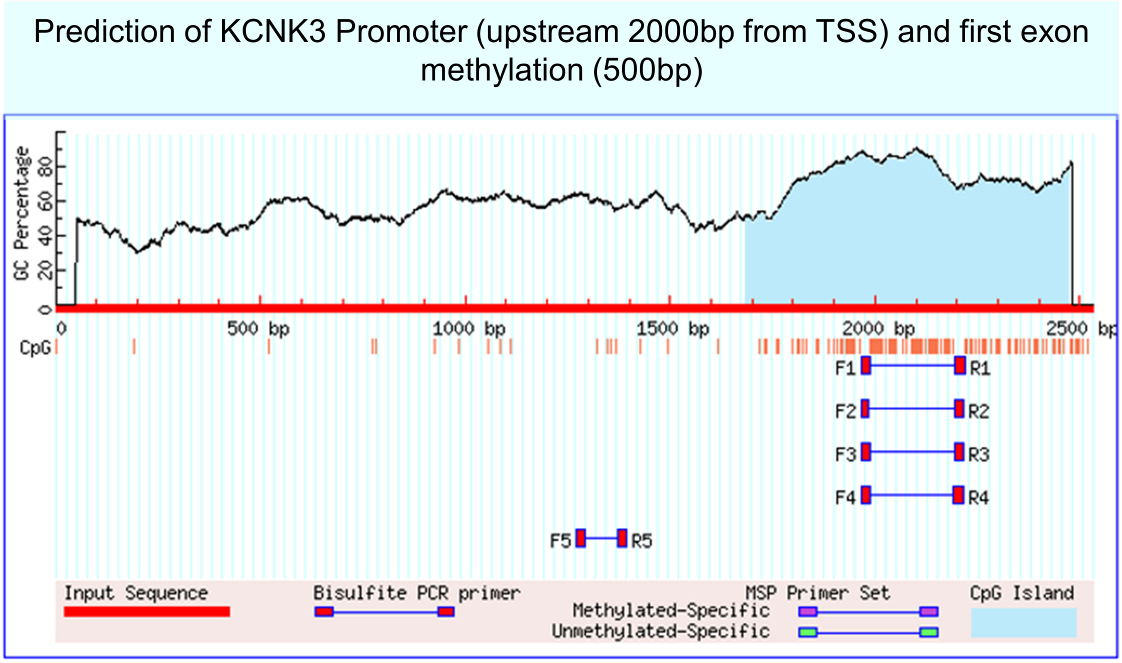

Supplementary Figure 4 Prediction of KCNK3 Promoter (upstream 2000bp from TSS) and first exon methylation (500bp) via MethPrimer (https://www.methprimer.com/)

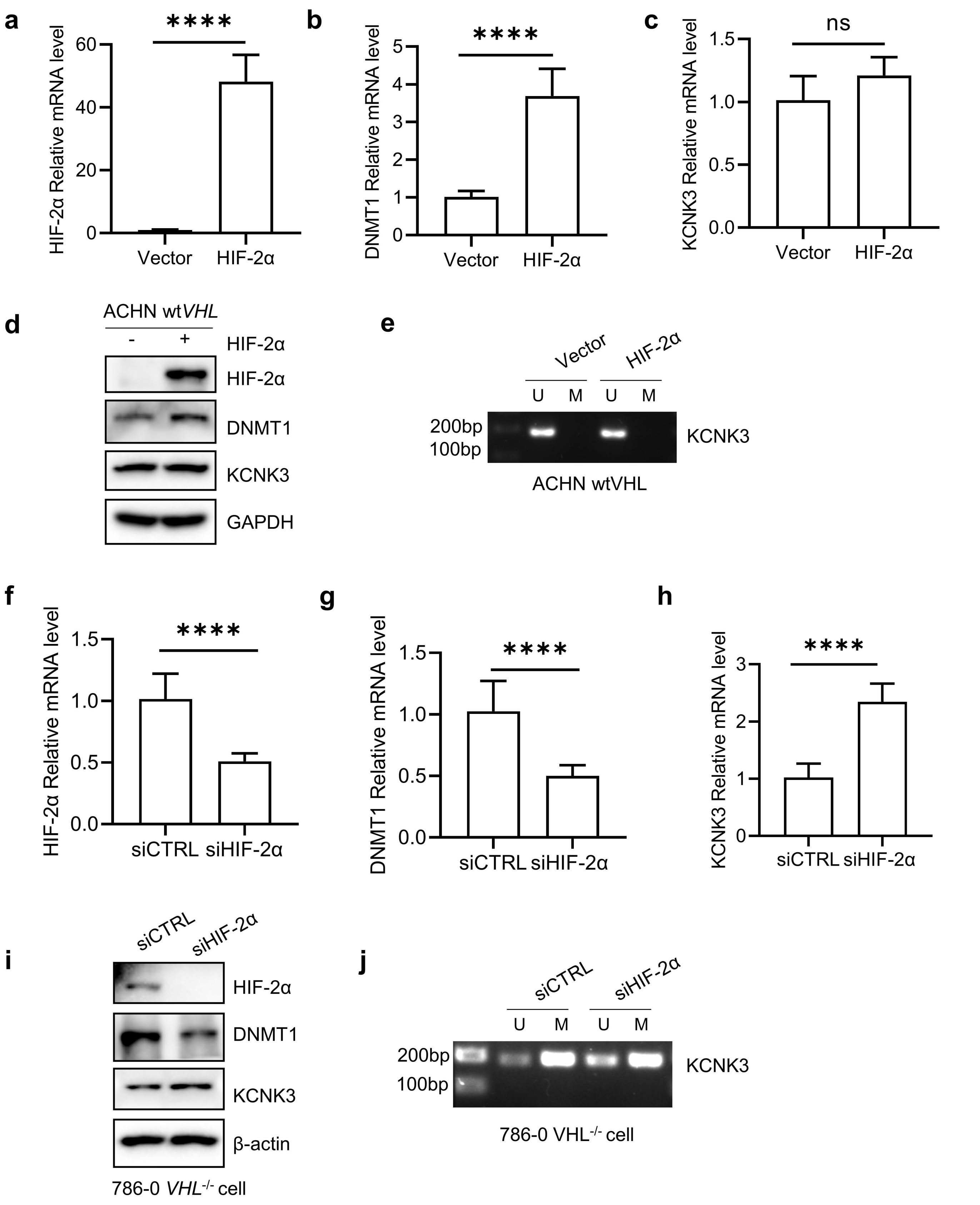

Supplementary Figure 5 a-e ACHN (wildtype VHL) cells were transfection with HIF-2α plasmid for 48 hours, the mRNA level (**a-c**) and protein level (**d**) of HIF-2α, DNMT1 and KCNK3 were assessed, and the methylation level of KCNK3 (**e**) were evaluated by MSP. **f-j** VHL-deficient 786-O cells were transfected with HIF-2α siRNA, the mRNA level (**f-h**) and protein level (**i**) of HIF-2α, DNMT1 and KCNK3 were assessed, and methylation level of KCNK3 were evaluated by MSP (**j**).

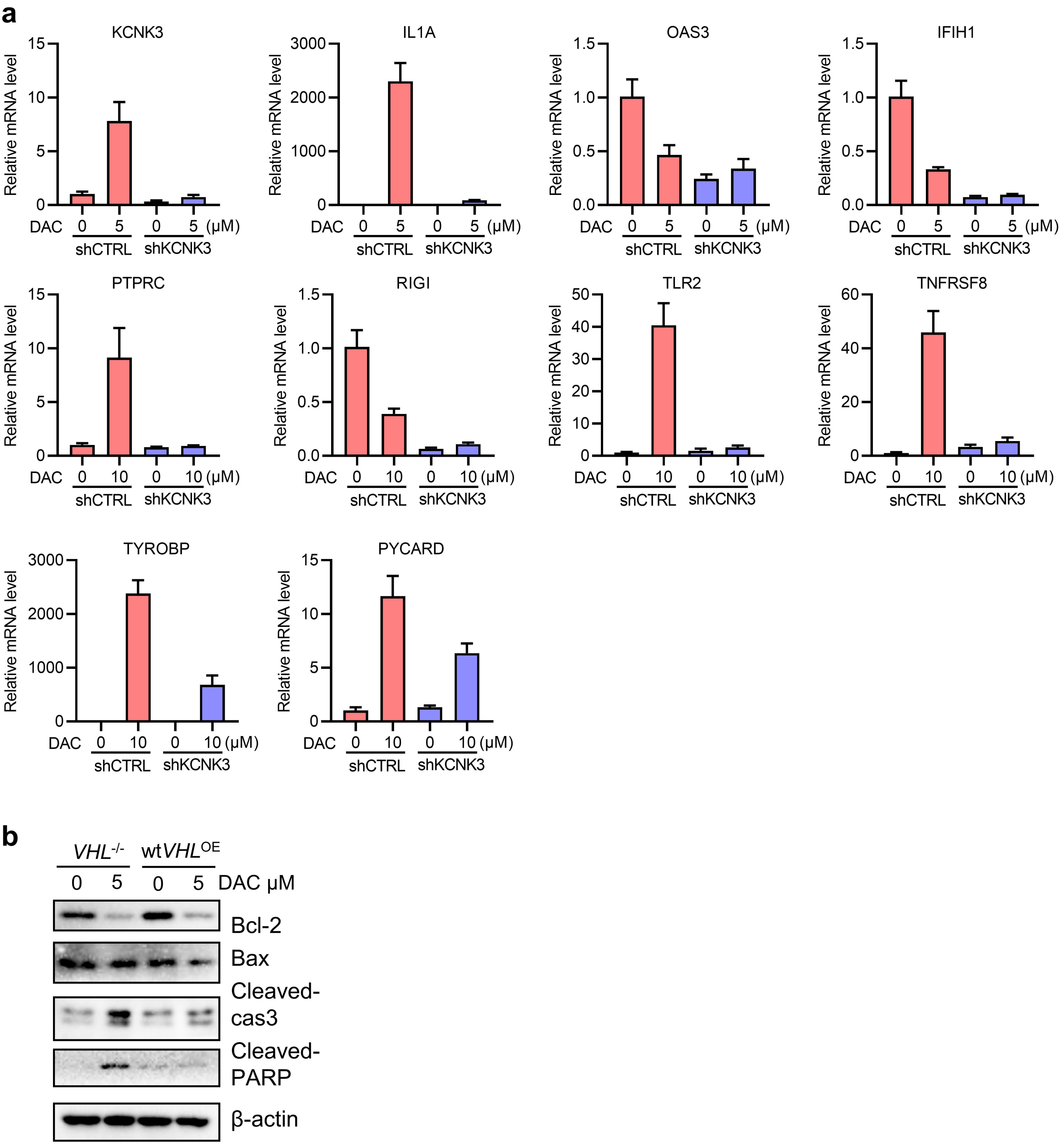

Supplementary Figure 6 a KCNK3 depletion reversed the decitabine induced-TNF-α production. 786-O shCTRL cells and shKCNK3 cells treated with or without decitabine (DAC), RT-qPCR analysis of mRNA level of the genes involved in Regulation of tumor necrosis factor production pathway. **b** Decitabine effect on apoptosis in 786-O VHL-isogenic cell pair. Western blotting showed the protein level of the genes involved in apoptosis pathway.

Supplementary Table 1 RCC patient information

Sample ID	Sample Source	Age	Gender	Pathology
RCC-2T	Biopsy	36	Male	Right kidney adrenal metastasis, consistent with low-grade renal cell carcinoma
RCC-5T	Surgery	61	Male	Right kidney middle, clear cell carcinoma Grade II (5*4*4 cm)
RCC-8T	Surgery	70	Male	Right kidney clear cell carcinoma Grade II (5.5*3.5*3.5 cm)

Supplementary Table 2 Antibodies used in this study.

Antibodies	Suppliers	Cat. No	Dilution for WB
VHL	Santa Cruz Biotechnology	sc-135657	1:1000
HA	Cell Signaling technology	3724	1:1000
HIF-2α	Santa Cruz Biotechnology	sc-13596	1:500
DNMT1	Santa Cruz Biotechnology	sc-57496	1:500
DNMT3A	Abcam	ab115730	1:1000
DNMT3B	Cell Signaling technology	2652	1:1000
BCL-2	Cell Signaling technology	3686	1:1000
BAX	Cell Signaling technology	2947S	1:1000
cleaved-caspase3	Cell Signaling technology	9664	1:1000
Cyclin D	Santa Cruz Biotechnology	sc-20044	1:500
KCNK3	Alomone labs	APC-024	1:1000
JNK	Cell Signaling technology	9258	1:1000
P-JNK	Cell Signaling technology	4668	1:1000
C-jun	Santa Cruz Biotechnology,	sc-1694	1:1000
P-c-jun	Cell Signaling technology	9164	1:1000
P38	Cell Signaling technology	9217	1:1000
P-p38	Cell Signaling technology	4511	1:1000
ERK	Cell Signaling technology	9102	1:1000
P-ERK	Cell Signaling technology	4370	1:1000
Cleaved-ARP	Cell Signaling technology	5625	1:1000
GAPDH	Santa Cruz Biotechnology	sc-47724	1:1000
β-actin	Santa Cruz Biotechnology,	sc-47778	1:1000

Supplementary Table 3 Sequences of siRNAs used in this study

	Gene name		Sequences (5'->3')
1	VHL siRNA1	Sense strand	AGGCAGGCGUCGAAGAGUATT
		Antisense strand	UACUCUUCGACGCCUGCCUTT
2	VHL siRNA2	Sense strand	GGCUCAACUUCGACGGCGATT
		Antisense strand	UCGCCGUCGAAGUUGAGCCTT
3	VHL siRNA3	Sense strand	GUGCUGCGCUCGGUGAACUTT
		Antisense strand	AGUUCACCGAGCGCAGCACTT
4	EPAS1 siRNA	Sense strand	AGUGCAUCAUGUGUGUCAACU
		Antisense strand	UUGACACACAUGAUGCACUGG
5	DNMT1 siRNA	Sense strand	CGAAUUUCUGCAAACAGAAAU
		Antisense strand	UUCUGUUUGCAGAAAUUCGUG
6	DNMT3A siRNA	Sense strand	CAGUGGUGUGUGUUGAGAAGC
		Antisense strand	UUCUCAACACACACCACUGAG
7	DNMT3B siRNA	Sense strand	CGAGGUCUCUGCAGACAAACU
		Antisense strand	UUUGUCUGCAGAGACCUCGGA
8	KCNK3 siRNA1	Sense strand	GUUUGCAUCUCUAUUUAUACC
		Antisense strand	UAUAAAUAGAGAUGCAAACGU
9	KCNK3 siRNA2	Sense strand	CCAUAAUUGCUGAUAAUUACC
		Antisense strand	UAAUUAUCAGCAAUUAUGGUG
10	KLF4 siRNA1	Sense strand	CAAGUUAUAUUUAAUCCAAAG
		Antisense strand	UUGGAUUAAAUAUAACUUGGA
11	KLF4 siRNA2	Sense strand	CUAUCAUUCCAAUUCUAAAUC
		Antisense strand	UUUAGAAUUGGAAUGAUAGAA

Supplementary Table 3 Continued. Sequences of siRNAs used in this study

	Gene name		Sequences (5'->3')
12	HTATIP2 siRNA1	Sense strand	GAUCGUUACUCUGUAUUUAGG
		Antisense strand	UAAAUACAGAGUAACGAUCAA
13	HTATIP2 siRNA2	Sense strand	CAUGUAAUUGUGUAAGUAAAC
		Antisense strand	UUACUUACACAAUUACAUGUA
14	SOX9 siRNA1	Sense strand	CUUCCUUAAAGACAUUUAAGC
		Antisense strand	UUAAAUGUCUUUAAGGAAGAA
15	SOX9 siRNA2	Sense strand	GUAUGUACUGUGUAUGAUUCA
		Antisense strand	AAUCAUACACAGUACAUACUA
16	HDAC4 siRNA1	Sense strand	GAUGAAGUUACAAGAAUUUGU
		Antisense strand	AAAUUCUUGUAACUUCAUCUU
17	HDAC4 siRNA2	Sense strand	GCUUUAUUGCUGUUUAAGAAA
		Antisense strand	UCUUAAACAGCAAUAAAGCAA
18	TBX3 siRNA1	Sense strand	GAAUGUUUCCUCCAUUUAAAG
		Antisense strand	UUAAAUGGAGGAAACAUUCGC
19	TBX3 siRNA2	Sense strand	GCAUUAUCGGUUCUCAAUAAA
		Antisense strand	UAUUGAGAACCGAUAAUGCAA
20	INHBA siRNA1	Sense strand	GCUCUUACCACAUGAUACAAG
		Antisense strand	UGUAUCAUGUGGUAAGAGCUG
21	INHBA siRNA2	Sense strand	GAUGACAUCAGUUGUUUAAAC
		Antisense strand	UUAAACAACUGAUGUCAUCAG
22	siControl siRNA	Sense strand	UUCUCCGAACGUGUCACGUTT
		Antisense strand	ACGUGACACGUUCGGAGAATT

Supplementary Table 3 Continued. Sequences of siRNAs used in this study

	Gene name		Sequences (5'->3')
23	KLF5 siRNA1	Sense strand	CUUACAGUAUCAACAUGAACG
		Antisense strand	UUCAUGUUGAUACUGUAAGGU
24	KLF5 siRNA2	Sense strand	GGAAUACAUUGUAUUAAUACC
		Antisense strand	UAUUAAUACAAUGUAUUCCCU
25	G0S2 siRNA1	Sense strand	GGAAGAUGGUGAAGCUGUACG
		Antisense strand	UACAGCUUCACCAUCUUCCCC
26	G0S2 siRNA2	Sense strand	CACUGUGUGAAUUAUCUAAAU
		Antisense strand	UUAGAUAAUUCACACAGUGUU
27	ASS1 siRNA1	Sense strand	GGUUUGAGCUCAGCUGCUACU
		Antisense strand	UAGCAGCUGAGCUCAAACCGG
28	ASS1 siRNA2	Sense strand	GGCGCUAAUUGUUGUGAUAAU
		Antisense strand	UAUCACAACAAUUAGCGCCUG
29	ATF3 siRNA1	Sense strand	GCAAAGUGCCGAAACAAGAAG
		Antisense strand	UCUUGUUUCGGCACUUUGCAG
30	ATF3 siRNA2	Sense strand	GCAUUAUUGGAUGUCAAUAGC
		Antisense strand	UAUUGACAUCCAAUAAUGCUA
31	COL6A3 siRNA1	Sense strand	GUAUCGUACUAAACAAGAAGU
		Antisense strand	UUCUUGUUUAGUACGAUACGU
32	COL6A3 siRNA2	Sense strand	CAAUAUUCGUGUUGGUUUAGU
		Antisense strand	UAAACCAACACGAAUAUUGUC
33	MMP2 siRNA1	Sense strand	CCCUAAAGAGAUACUUUGAUA
		Antisense strand	UCAAAGUAUCUCUUUAGGGGU

Supplementary Table 3 Continued. Sequences of siRNAs used in this study

	Gene name		Sequences (5'->3')
34	MMP2 siRNA2	Sense strand	GGGAAAUGUCAACAAGUAUGA
		Antisense strand	GAGGAAUUGAGAACUACUAUG
35	TSPAN15 siRNA1	Sense strand	UAGUAGUUCUCAAUUCCUCUU
		Antisense strand	GAGUUUCUGACUAAUCAAAGC
36	TSPAN15 siRNA2	Sense strand	UUUGAUUAGUCAGAAACUCCU
		Antisense strand	AUACUUGUUGACAUUUCCCAU

Supplementary Table 4 The sequence information of the RT-qPCR primers used in this study

No.	Gene name	Suppliers		Sequences (5'->3')
1	GAPDH	BGI	Forward	ACAACTTTGGTATCGTGGAAGG
		BGI	Reverse	GCCATCACGCCACAGTTTC
2	VHL	BGI	Forward	GGAGCCTAGTCAAGCCTGAGA
		BGI	Reverse	CATCCGTTGATGTGCAATGCG
3	DNMT1	BGI	Forward	AGAACGGTGCTCATGCTTACA
		BGI	Reverse	CTCTACGGGCTTCACTTCTTG
4	DNMT3A	BGI	Forward	CCGATGCTGGGGACAAGAAT
		BGI	Reverse	CCCGTCATCCACCAAGACAC
5	DNMT3B	BGI	Forward	AGGGAAGACTCGATCCTCGTC
		BGI	Reverse	GTGTGTAGCTTAGCAGACTGG
6	TET	BGI	Forward	CATCAGTCAAGACTTTAAGCCCT
		BGI	Reverse	CGGGTGGTTTAGGTTCTGTTT
7	TET2	BGI	Forward	GATAGAACCAACCATGTTGAGGG
		BGI	Reverse	TGGAGCTTTGTAGCCAGAGGT
8	TET3	BGI	Forward	TCCAGCAACTCCTAGAACTGAG
		BGI	Reverse	AGGCCGCTTGAATACTGACTG
9	KCNK3	BGI	Forward	CTACGAGCACTGGACCTTCTT
		BGI	Reverse	CGTAAGGATGTAGACGAAGCTGA
10	KLF4	BGI	Forward	CAGCTTCACCTATCCGATCCG
		BGI	Reverse	GACTCCCTGCCATAGAGGAGG
11	HTATIP2	BGI	Forward	CGGAGGGATTTGTTCGTGTTG
		BGI	Reverse	AGCTCCTTTAGAGGATAGCAAGT

Supplementary Table 4 Continued. The sequence information of the RT-qPCR primers used in this study

No.	Gene name	Suppliers		Sequences (5'->3')
12	SOX9	BGI	Forward	AGCGAACGCACATCAAGAC
		BGI	Reverse	CTGTAGGCGATCTGTTGGGG
13	HDAC4	BGI	Forward	AGCGTCCGTTGGATGTCAC
		BGI	Reverse	CCTTCTCGTGCCACAAGTCT
14	TBX3	BGI	Forward	CCCGGTTCCACATTGTAAGAG
		BGI	Reverse	GTATGCAGTCACAGCGATGAAT
15	INHBA	BGI	Forward	CCTCCCAAAGGATGTACCCAA
		BGI	Reverse	CTCTATCTCCACATACCCGTTCT
16	KLF5	BGI	Forward	CCTGGTCCAGACAAGATGTGA
		BGI	Reverse	GAACTGGTCTACGACTGAGGC
17	G0S2	BGI	Forward	CGTGCCACTAAGGTCATTCC
		BGI	Reverse	GCACGTACAGCTTCACCATC
18	ASS1	BGI	Forward	CTTGGGGCCAAAAAGGTGTTC
		BGI	Reverse	GAGGTAGCGGTCCTCATACAG
19	ATF3	BGI	Forward	CCTCTGCGCTGGAATCAGTC
		BGI	Reverse	TTCTTTCTCGTCGCCTCTTTTT
20	COL6A3	BGI	Forward	CTGTTCCTCTTTGACGGCTCA
		BGI	Reverse	CCTTGACATCATCGCTGTACTGA
21	MMP2	BGI	Forward	GATACCCCTTTGACGGTAAGGA
		BGI	Reverse	CCTTCTCCCAAGGTCCATAGC
22	TSPAN15	BGI	Forward	CCGCCACAGCACTTGAACT
		BGI	Reverse	CCGCCACAGCACTTGAACT

Supplementary Table 4 Continued. The sequence information of the RT-qPCR primers used in this study

No.	Gene name	Suppliers		Sequences (5'->3')
23	TNF-α primer1	BGI	Forward	CCTCTCTCTAATCAGCCCTCTG
		BGI	Reverse	GAGGACCTGGGAGTAGATGAG
24	TNF-α primer2	BGI	Forward	GAGGCCAAGCCCTGGTATG
		BGI	Reverse	CGGGCCGATTGATCTCAGC
25	IL1A	BGI	Forward	TGGTAGTAGCAACCAACGGGA
		BGI	Reverse	ACTTTGATTGAGGGCGTCATTC
26	OAS3	BGI	Forward	CCTGATTCTGCTGGTGAAGCAC
		BGI	Reverse	TCCCAGGCAAAGATGGTGAGGA
27	IFIH1	BGI	Forward	TCGAATGGGTATTCCACAGACG
		BGI	Reverse	GTGGCGACTGTCCTCTGAA
28	PTPRC	BGI	Forward	ACCACAAGTTTACTAACGCAAGT
		BGI	Reverse	TTTGAGGGGGATTCCAGGTAAT
29	RIGI	BGI	Forward	CTGGACCCTACCTACATCCTG
		BGI	Reverse	GGCATCCAAAAAGCCACGG
30	TLR2	BGI	Forward	AACTTACTGGGAAATCCTTAC
		BGI	Reverse	AAAAATCTCCAGCAGTAAAAT
31	TNFRSF8	BGI	Forward	TCCACGGAGCACACCAATAAC
		BGI	Reverse	ACTGAGAGCATGACATCGCTG
32	TYROBP	BGI	Forward	ACTGAGACCGAGTCGCCTTAT
		BGI	Reverse	ATACGGCCTCTGTGTGTTGAG
33	PYCARD	BGI	Forward	TGGATGCTCTGTACGGGAAG
		BGI	Reverse	CCAGGCTGGTGTGAAACTGAA

Supplementary Table 5 Bisulfite sequencing PCR (BSP) Primers of KCNK3 used in this study

No.	Primer	Suppliers		Sequences (5'->3')
1	First round primer	BGI	Forward	AGGAGGGYGGTGGGTGGTGT
	First round primer	BGI	Reverse	CCCAACCCTAAATTCCCRCCC
2	Second round primer	BGI	Forward	TGGTGTTGAAGGGATAGTTT
	Second round primer	BGI	Reverse	ATAATAACRAAATAAAAAAAACC

Supplementary Table 6 Methylation-specific PCR (MSP) Primers of KCNK3 used in this study

No.	Primer	Suppliers		Sequences (5'->3')
1	M primer	BGI	Forward	TATTTTTATTTATTTGTTGGTGGGC
	M primer	BGI	Reverse	CCGACCTTATACGACTTAAAACG
2	U primer	BGI	Forward	TTTTTATTTATTTGTTGGTGGGTGT
	U primer	BGI	Reverse	CCAACCTTATACAACTTAAAACACA
